# Clinically Important Emerging and Uncommon Fungal Pathogens in Asia: Epidemiology, Antifungal Resistance and Challenges in Diagnosis and Management

**DOI:** 10.1111/myc.70222

**Published:** 2026-09-08

**Authors:** Narut Chancharussin, Anupop Jitmuang, Arunaloke Chakrabarti, Methee Chayakulkeeree

**Affiliations:** ^1^ Division of Infectious Diseases and Tropical Medicine, Department of Medicine, Faculty of Medicine Siriraj Hospital Mahidol University Bangkok Thailand; ^2^ Department of Medical Microbiology Postgraduate Institute of Medical Education and Research Chandigarh India

**Keywords:** antifungal resistance, basidiomycetes, *Candida*, mould, mucormycosis, oomycetes, rare fungal infection, yeast

## Abstract

Fungal infections in Asia are influenced by warm, humid climates, high population density and expanding healthcare systems, which together facilitate the emergence, transmission and detection of both common and rare pathogens. While *Candida* and *Aspergillus* species remain the leading causes of invasive fungal disease, increasing attention has focused on uncommon yeasts and rare moulds, many of which show intrinsic or acquired antifungal resistance. Advances in molecular diagnostics and susceptibility testing have improved identification of emerging pathogens, including uncommon *Candida* species and rare moulds such as Mucorales, filamentous Basidiomycetes, non‐*marneffei Talaromyces*, Oomycetes and thermally dimorphic fungi like *Emergomyces*. These organisms primarily affect immunocompromised individuals and present significant diagnostic and therapeutic challenges due to identification difficulties, variable susceptibility patterns and limited clinical experience. Clinical presentations range from fungaemia and invasive pulmonary disease to cutaneous and disseminated infections. Treatment is guided by pathogen and susceptibility profiles, commonly involving echinocandins, amphotericin B and azoles. In some cases, particularly infections caused by Oomycetes and Mucorales, prompt surgical intervention is essential. This review provides a comprehensive overview of current epidemiological trends, risk factors, clinical features, antifungal susceptibility patterns and treatment outcomes associated with rare and emerging fungal pathogens in Asia. By highlighting these increasingly recognized infections, it aims to enhance awareness among clinicians and mycologists, promote evidence‐based diagnostic and therapeutic approaches and inform future research addressing this evolving and clinically significant public health challenge.

## 1 Introduction

Fungal infections in Asia are strongly shaped by environmental and ecological factors. Warm, humid climates across the region promote fungal growth and spore dispersal, while rapid urbanization and high population density increase exposure to airborne fungi and facilitate the spread of antifungal‐resistant species [1, 2, 3, 4]. These conditions contribute to the high prevalence and diversity of fungal diseases. The epidemiology varies: South Asia faces a high disease burden due to tropical climates, a high burden of patients in public sector hospitals and healthcare gaps; East Asia is seeing rising cases linked to urbanization and more immunocompromised individuals; and Southeast Asia's biodiversity and seasonal climate shifts allow a wide range of fungal pathogens to emerge, including regionally endemic species such as *Talaromyces marneffei* and uncommon yeasts and moulds [5, 6, 7, 8, 9].

While *Candida* and *Aspergillus* remain the most common causes of invasive fungal disease, reports of rare yeasts and moulds as aetiologic agents are steadily increasing, particularly among immunocompromised hosts [10, 11]. Advances in molecular identification (e.g., genomic sequencing, matrix‐assisted laser desorption/ionization time‐of‐flight mass spectrometry [MALDI‐TOF MS], PCR) and wider use of fungal susceptibility testing have revealed the clinical importance of uncommon yeasts such as *Candida kefyr*, *Pichia* (*Candida*) *norvegensis* and *Candidozyma* (*Candida*) *auris*, many of which show intrinsic or acquired antifungal resistance [12, 13, 14]. Likewise, rare moulds, including newly emerged species of Mucorales, filamentous Basidiomycetes, non‐*marneffei* species of *Talaromyces* and other unusual filamentous fungi, are being recognized with growing frequency, causing infections particularly in immunocompromised populations [15, 16, 17]. The emergence of these rare fungal pathogens presents significant diagnostic and therapeutic challenges, as emphasized in recent global and regional guidelines for non‐*Aspergillus* moulds [10, 11]. Limited clinical experience, variable antifungal susceptibility patterns and the lack of standardized treatment guidelines complicate patient management [18, 19]. For clinicians and mycologists across Asia, these infections represent a growing concern that requires greater recognition, improved diagnostics and further study.

This review summarizes the current knowledge of emerging and uncommon yeasts and moulds of clinical importance emerging in Asia. We highlight epidemiological trends, documented risk factors, clinical manifestations, diagnostic methods, antifungal susceptibility profiles where available and reported treatment outcomes, with the aim of supporting clinicians and researchers in addressing this evolving threat.

## 2 Materials and Methods

### 2.1 Literature Search Strategy

A systematic literature search was performed in PubMed to identify reports of emerging and uncommon fungal pathogens in Asia. The search was restricted to articles published in English between 1 January 2020 and 31 December 2025. Eligible publication types included case reports, case series, narrative reviews, systematic reviews, meta‐analyses and relevant books or guideline documents. The search strategy incorporated combinations of the following terms: “emerging fungal infections,” “neglected fungal pathogens,” “rare yeast infections,” “uncommon yeast infections,” “emerging yeast infections,” “non‐ *Candida albicans* ,” “rare molds,” “uncommon mold infections,” “filamentous fungal infections,” “opportunistic fungal infections,” “Basidiomycetes infections,” “fungal‐like,” “Oomycetes infections” and “oomycoses.” The reference lists of eligible articles were also manually reviewed to identify additional relevant publications. Duplicate records were removed before screening.

### 2.2 Study Screening and Pathogen Selection

**FIGURE 1:**
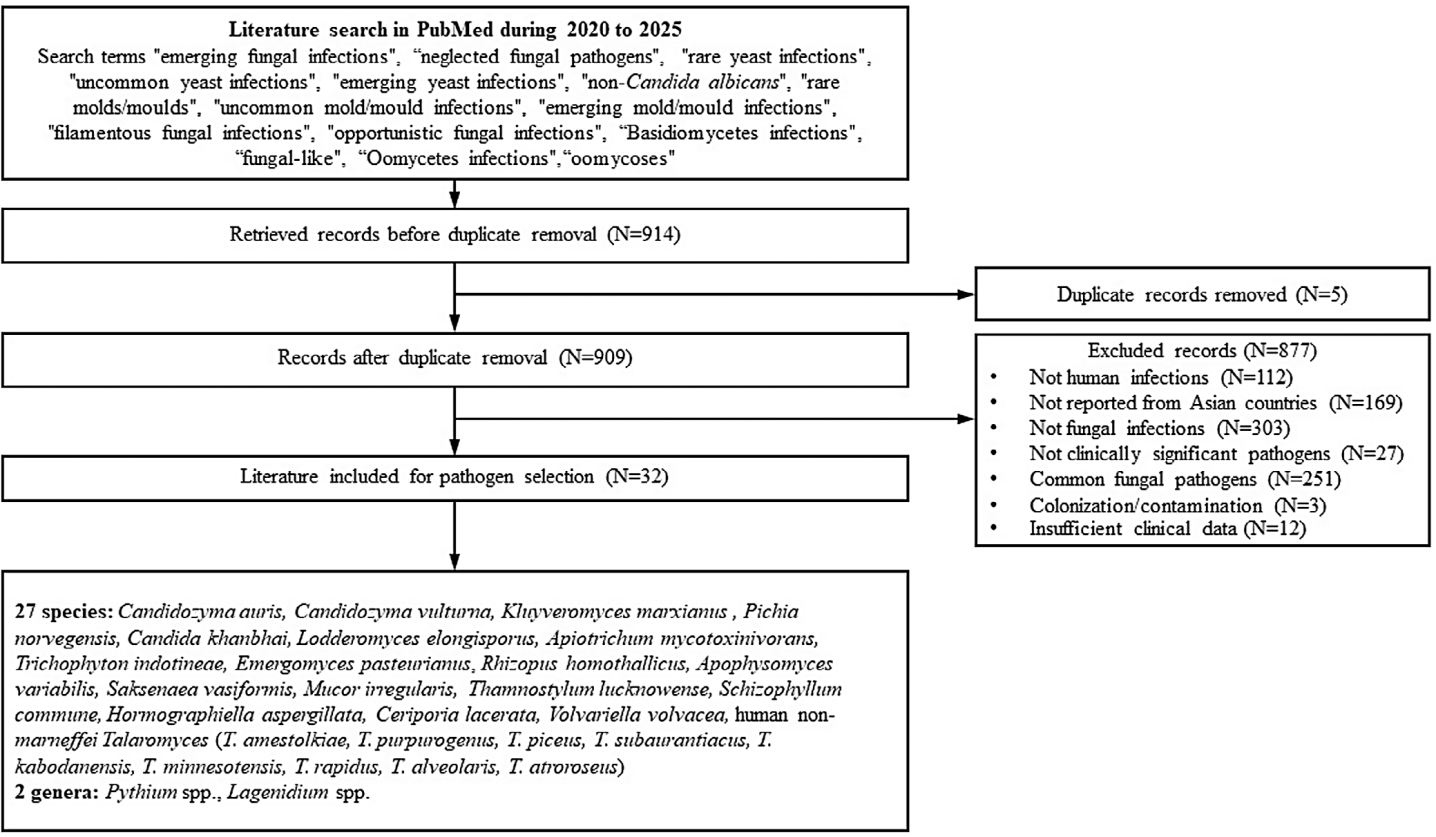
Flow chart of literature screening and selection process.

After initial screening, duplicate articles and studies unrelated to human fungal infections in Asia were excluded. For the purpose of this review, ‘emerging’ fungal pathogens were defined as organisms demonstrating increasing recognition in clinical practice, expanding geographic distribution, newly recognized pathogenicity or evolving diagnostic and antifungal resistance challenges, rather than solely representing newly discovered species [20]. The remaining articles were reviewed to identify fungal pathogens that fulfilled the predefined pathogen selection criteria. Pathogens were eligible for inclusion if they were yeasts, moulds or fungal‐like organisms causing proven or probable human infection, were reported predominantly from Asian countries or demonstrated increasing recognition in Asia during the study period (2020–2025), and fulfilled at least one of the following criteria: (i) association with unusual clinical syndromes, novel host populations, outbreaks or high mortality; (ii) diagnostic challenges requiring advanced phenotypic or molecular identification methods beyond conventional microbiological techniques; or (iii) intrinsic or emerging antifungal resistance with potential therapeutic implications. To maintain the review's focus on under‐recognized pathogens, well‐established fungal pathogens with extensive clinical evidence and dedicated international guidelines, including common species of *Candida* and *Aspergillus*, as well as members of the order Mucorales, were excluded. Organisms with insufficient evidence supporting pathogenicity in humans were also excluded. In addition, pathogens with inadequate published data regarding clinical manifestations, antifungal susceptibility, treatment or outcomes were not considered suitable for detailed review. The study selection process is summarized in Figure 1. Using these predefined criteria, 27 fungal species representing 29 clinically relevant taxa were selected from 32 eligible publications for detailed review (Figure 1).

### 2.3 Data Extraction and Synthesis

For each selected pathogen, available data regarding epidemiology, geographic distribution, host characteristics, clinical manifestations, diagnostic methods, antifungal susceptibility patterns, treatment approaches and clinical outcomes were extracted and summarized. Information from additional relevant publications identified through reference screening was incorporated when appropriate to provide broader clinical context and ensure comprehensive coverage. The extracted data were synthesized narratively, with emphasis on the epidemiology of these pathogens in Asia, emerging trends, diagnostic challenges, antifungal resistance and implications for clinical management.

## 3 Results and Discussions

In this review, we identified and summarized clinically significant emerging and uncommon yeasts and moulds in Asia, including *Candidozyma* (*Candida*) *auris*, *Candidozyma* (*Candida*) *vulturna*, *Kluyveromyces marxianus*, *Pichia norvegensis*, *Candida khanbhai*, *Lodderomyces elongisporus*, *Apiotrichum mycotoxinivorans*, *Trichophyton indotineae*, newly emerging Mucorales species, non‐*marneffei Talaromyces* species, filamentous basidiomycetes and *Emergomyces* species.

### 3.1 Rare Yeasts

#### 3.1.1 *Candidozyma* (*Candida*) *auris*

*Candidozyma auris* was first identified in 2009 from the ear canal of a patient in Japan, hence the name ‘auris’, derived from the Latin word for ‘ear’ [21]. Whole‐genome sequencing of clinical *C. auris* strains has revealed six geographically distinct clades with differing virulence profiles [22, 23, 24]. Four of these clades originated in Asian countries (Bangladesh, India, Iran, Japan, Pakistan and Singapore) [23, 24]. However, the geographic attribution of individual clades should be interpreted cautiously because it is based largely on the locations where isolates were first recognized. Subsequent international transmission may obscure the true origins and current distribution of these clades. Importantly, *C. auris* exhibits clade‐dependent antifungal susceptibility [23, 24]. Clade II (East Asian) is typically susceptible to all major antifungal classes and predominantly causes ear infections, whereas Clades I, III and IV are frequently multidrug‐resistant, particularly in nosocomial outbreaks [23, 24]. Unlike other *Candida* species, *C. auris* preferentially colonizes human skin and non‐living surfaces, including medical devices, rather than the gastrointestinal tract [25, 26]. Microbiologically, it is a budding yeast closely related to *C. haemulonii* and *C. duobushaemulonii* [27]. Unlike *C. albicans* , *C. auris* rarely forms pseudohyphae in general conditions [28]. It cannot be reliably distinguished from other *Candida* species by conventional methods. However, it grows well at elevated temperatures (40°C–42°C) [21], produces smooth, white‐to‐cream‐coloured colonies [21], and the development of a new chromogenic medium (CHROMagar Candida Plus) enables rapid identification and differentiation from other species [29]. A recent discovery of a *C. auris* ‐specific adhesin, surface colonization factor 1 (Scf1), has highlighted its role in biofilm formation, long‐term colonization of skin and medical devices and virulence during invasive infection [30]. Interestingly, isolates from certain clades show increased susceptibility to UV‐C light, suggesting new possibilities for disinfection strategies in healthcare environments [31]. The most common mechanism of azole resistance in *C. auris* involves mutations in the *ERG11* gene, which encodes the drug target lanosterol 14α‐demethylase [32, 33, 34]. Echinocandins are recommended as empirical or initial therapy, with micafungin preferred over caspofungin due to its lower minimum inhibitory concentrations (MICs) [35, 36]. Although resistance to echinocandins remains uncommon (around 5%), it may develop during treatment and is typically associated with *FKS1* mutations [32, 33, 34]. Routine combination therapy is not currently recommended but may be considered on a case‐by‐case basis [37]. For example, liposomal amphotericin B combined with echinocandins has shown a synergistic effect, reducing the MIC of micafungin by up to 64‐fold in 8 of 10 isolates [38]. Additionally, in vitro studies indicate that combining posaconazole with protease inhibitors such as atazanavir or saquinavir enhances fungistatic activity and significantly reduces *C. auris* biofilm formation [39]. These findings are hypothesis‐generating but should be interpreted cautiously, as in vitro synergy does not necessarily translate into improved clinical outcomes. The small numbers of isolates studied, variation in synergy‐testing methods, potential toxicity, drug interactions and lack of controlled clinical data currently limit the applicability of these combinations in routine practice. Well‐designed multicentre clinical studies, supported by standardized susceptibility testing and genomic surveillance, are needed to define optimal treatment strategies.

#### 3.1.2 *Candidozyma* (*Candida*) *vulturna*

*Candidozyma vulturna*, a recently recognized member of the *C. haemulonii* complex and closely related to *C. auris* , has emerged as an opportunistic human pathogen in Asia [40, 41, 42]. It was first isolated from flowers in Mindanao, the Philippines, in 2016 [43] and subsequently reported to cause bloodstream infections [44, 45]. Conventional identification methods, including VITEK2 and MALDI‐TOF, often misidentify *C. vulturna* as *C. haemulonii*, complicating accurate diagnosis [41, 42]. The species exhibits intrinsic resistance to azoles, with additional resistance observed to amphotericin B, fluconazole and voriconazole, while remaining susceptible to echinocandins such as anidulafungin and caspofungin [41, 42]. A recent narrative review [41] documented 94 cases over the past decade, including 3 clonal outbreaks in Brazil, Vietnam and China, with subsequent spread across multiple continents. Infections mainly affect males from Asia and Latin America, with typical risk factors for candidaemia, with an overall mortality of 18%. Phylogenetic analyses confirm *C. vulturna*'s close relationship to *C. auris* and *C. haemulonii*, suggesting shared pathogenic traits [41]. Notably, *C. vulturna* demonstrates significant biofilm formation capabilities, relatively more than those in *C. auris* and *C. haemulonii*, contributing to its persistence in clinical environments [42]. In Malaysia, among 19 reported cases, 47% originated from Sabah, with additional cases from Klang Valley, Sarawak and other states, highlighting its regional clinical significance and underscoring the importance of susceptibility‐guided antifungal therapy [46]. Whether *C. vulturna* represents a genuinely emerging pathogen or has historically been under‐recognized remains uncertain, as earlier cases were likely misidentified because of the limited discriminatory ability of conventional diagnostic methods. The current understanding of *C. vulturna* remains constrained by the rarity of reported cases, reliance on retrospective observational studies and the absence of prospective multicentre cohorts.

#### 3.1.3 *Kluyveromyces marxianus* (*Candida kefyr*)

*Candida kefyr*, whose teleomorph is classified as *Kluyveromyces marxianus* [47], was first isolated in 1909 from kefir, a fermented milk beverage [48]. Although it remains a rare cause of human disease, recent reports suggest that it may be an emerging pathogen, particularly among immunocompromised patients [48]. Cases have been described in individuals with haematologic malignancies, including allogeneic haematopoietic stem cell transplant recipients, in whom infection typically occurs 14–300 days post‐transplantation despite antifungal prophylaxis, as well as in kidney transplant recipients who did not receive prophylaxis [48]. *Candida kefyr* is notable for its ability to form biofilms and its high propensity to cause infection through colonization [49]. Antifungal susceptibility testing shows that most isolates are sensitive to commonly tested agents, with favourable responses observed to both azoles and amphotericin B, although reports of resistance have also been documented [48]. Compared with candidaemia caused by other *Candida* species, *C. kefyr* infection is generally associated with better outcomes and a lower mortality rate [48]. However, these observations should be interpreted cautiously because published studies include relatively few patients, often lack appropriate comparator groups and are susceptible to selection and publication bias.

#### 3.1.4 *Pichia* (*Candida*) *norvegensis*

Recently reclassified as *Pichia norvegensis* [50], *Candida norvegensis* was first described in 1954 after being isolated from the sputum of three asthmatic patients in Norway [51]. Although infections caused by this species are rare, several potential risk factors have been reported, including human immunodeficiency virus (HIV) infection, which is associated with invasive and oropharyngeal candidiasis, and malignancies such as hepatocellular carcinoma, which have also been linked to invasive disease [52, 53, 54]. In addition to invasive candidiasis, *P. norvegensis* peritonitis has been documented in a renal transplant patient undergoing peritoneal dialysis [55], highlighting its potential to cause opportunistic infections in immunocompromised hosts. Although *P. norvegensis* has only recently gained clinical recognition, a study published in 2010 analysing data from 1997 to 2007 demonstrated a 5‐ to 10‐fold increase in the rate of *P. norvegensis* isolation over the study period, suggesting an increasing clinical relevance of this uncommon yeast [56].

Antifungal susceptibility profiles vary across published studies. Overall, *P. norvegensis* is resistant to fluconazole and flucytosine [57, 58]. In contrast, susceptibility to voriconazole and echinocandins has been inconsistent, with variable results reported among different isolates and studies [58, 59]. Amphotericin B has traditionally been regarded as the treatment of choice [58, 60], despite limited supporting evidence from the small number of documented infections. More recently, several reports have demonstrated susceptibility of *P. norvegensis* isolates to echinocandins [56, 61, 62, 63], suggesting these agents as potential therapeutic alternatives.

#### 3.1.5 *Candida khanbhai*

Named in honour of Prof. Dr. Ziauddin Khan (with ‘bhai’ meaning ‘friend’ or ‘brother’ in Hindi), it has only two reported clinical isolates to date [64]. The first was obtained from nasal colonization of a patient during a *C. auris* nosocomial outbreak in Kuwait in 2019, initially misidentified as *C. duobushaemulonii* by VITEK 2 (bioMérieux) with 99% probability [64]. The second was isolated in 2014 from the blood culture of a 55‐year‐old man with no known comorbidities, who had been hospitalized for the treatment of new‐onset hospital‐acquired pneumonia in Malaysia. This isolate was biochemically identified as *Clavispora* (*Candida*) *lusitaniae* by API 20C (bioMérieux) with 81% probability. Despite starting 400 mg/day of intravenous fluconazole on Day 12 of admission, the patient's condition deteriorated, and he died [64]. Subsequent sequence‐based analysis using the ITS1‐5.8S‐ITS2 region and the D1/D2 domain of the large subunit rDNA confirmed these isolates as a distinct lineage basal to the *C. haemulonii* species complex, which includes the clinically relevant species *C. duobushaemulonii*, *C. pseudohaemulonii* and *C. vulturna* [64]. Conventional laboratory methods often misidentify isolates, underscoring the need for advanced diagnostic approaches. Phenotypically, colonies were off‐white, glossy, soft and butyrous with entire margins. Cells were ovoid to spherical (1.5–3.5 × 2.5–4.5 μm), occurring singly, in pairs or short chains, with monopolar budding and no pseudohyphae observed after up to 12 weeks of incubation [64]. On chromogenic agar (Candida Plus), colonies appeared pale cream to lavender, with a distinctive blue halo like that of *C. auris* . MALDI‐TOF MS failed to yield a definitive match, with the closest hits to *C. pseudohaemulonii* and *Clostridium cadaveris* [64]. Biochemically, isolates exhibited delayed but positive maltose fermentation, no growth in 50%–60% glucose, tolerance to 0.1% cycloheximide and growth at up to 42°C [64]. Antifungal susceptibility testing revealed high MICs to amphotericin B and azoles, underscoring its potential clinical significance and diagnostic challenges [64]. However, these observations are based on only two isolates and should not be interpreted as representative of the species as a whole.

#### 3.1.6 *Lodderomyces elongisporus*

First described as *Saccharomyces elongisporus* in 1952 [65], it is a homothallic fungus capable of sexual reproduction with multiple ascospore formation [66]. It has been isolated from soil, fermented food products, plants, stored apples, pigeon excreta, insects, marine fish and humans, and it can survive in hospital environments [67, 68, 69, 70, 71, 72], with reports of nosocomial infections, including an outbreak in a neonatal care unit [72]. To date, more than 40 human cases of *L. elongisporus* infection have been reported worldwide [73]. Most infections occur at the extremes of age, particularly in neonates and elderly patients [73]. Reported risk factors include malignancy (especially gastrointestinal cancers), while comorbid conditions such as heart disease, advanced malignancy and end‐stage renal disease have been associated with fatal outcomes in adults [73]. The most common clinical presentation is bloodstream infection, though invasive disease with oropharyngeal infections, endocarditis, osteomyelitis and meningitis [74, 75, 76] has also been documented. Its medical significance was first recognized in 2008 when a retrospective review of 542 clinical *Candida parapsilosis* isolates from 25 countries revealed that 10 were actually *L. elongisporus* [77]. This species closely resembles *C. parapsilosis* , displaying oval to elongated yeast cells or pseudohyphal forms, cream‐coloured colonies on Sabouraud dextrose agar and the ability to assimilate high‐molecular‐weight paraffins, which often leads to misidentification by conventional methods [77, 78]. ITS rDNA sequencing has been used to resolve such discrepancies, particularly when MALDI‐TOF identified *L. elongisporus* in isolates misclassified by RapID Yeast Plus as *C. parapsilosis* , *C. stellatoidea* or *Geotrichum* spp. [79]. Phylogenetic analyses show a slightly larger genome size (15–16 Mb) compared to *C. parapsilosis* (12–13 Mb), and it belongs to the CTG clade, where the CUG codon is translated as serine instead of leucine, along with other human pathogens such as *C. albicans* , *C. tropicalis* and *C. lusitaniae* [73, 80]. Distinguishing features include greater sensitivity to salinity, hydrogen peroxide and pH fluctuations, maintenance of the yeast form within phagocytes, and lower biofilm production (similar to *C. parapsilosis* ) than *C. albicans, C. tropicalis* and *C. dubliniensis*. However, reports of increased MICs to sodium hypochlorite and isolation from catheter tips suggest biofilm‐forming potential [81]. These characteristics indicate that *L. elongisporus* has various adaptive mechanisms that enable persistence and pathogenicity in clinical settings [81]. A 2023 case series reported antifungal susceptibility testing of *L. elongisporus*, demonstrating generally low MICs for most antifungal agents [82]. Notably, three patients received echinocandins to treat *L. elongisporus* fungaemia, with favourable outcomes [82]. These findings should be interpreted cautiously because treatment recommendations are based almost exclusively on observational data involving a small number of patients. No species‐specific clinical breakpoints or prospective therapeutic studies are available, and current management is largely extrapolated from guidelines for candidaemia caused by other *Candida* species.

#### 3.1.7 *Apiotrichum mycotoxinivorans*

*Apiotrichum mycotoxinivorans* was first described in 2004 and is classified within the Basidiomycetes [83]. Formerly designated *Trichosporon mycotoxinivorans*, it was reclassified in 2015 based on updated phylogenetic analyses [84]. The organism has been isolated from environmental sources, including soil and river water, as well as from farm animals [85]. It was initially recognized as an emerging pathogen causing pulmonary infections, particularly among patients with cystic fibrosis, with the first clinical report published in 2009 [86]. Subsequently, an increasing number of extrapulmonary infections have been documented in Thailand, China, India, Latin America, the United States and several European countries [87, 88, 89, 90]. Reported clinical manifestations include fungaemia in patients with end‐stage renal disease receiving haemodialysis [87], intra‐abdominal abscesses following pancreaticoduodenectomy [87] and meningitis in a patient with B‐cell acute lymphoblastic leukaemia after neurosurgical intervention [88, 89]. Notably, two cases of haemodialysis‐associated bloodstream infection with concomitant *Stenotrophomonas maltophilia* bacteraemia have been described [87], although the clinical significance of this association remains unclear. Therefore, routine empirical antibacterial therapy cannot be recommended solely on the basis of the currently available evidence.

Morphologically, *A. mycotoxinivorans* produces oval to cuboid yeast‐like cells forming arthroconidia arranged in a characteristic beaded pattern, and colonies typically display a milky appearance [91]. For laboratory identification, MALDI‐TOF MS has emerged as a reliable and rapid alternative to PCR‐based methods for identifying rare fungal pathogens [88]. Antifungal susceptibility testing generally reveals low MICs for voriconazole but elevated MICs for amphotericin B [87, 88, 89]. Voriconazole is therefore considered the treatment of choice for invasive *A. mycotoxinivorans* infections [87, 88], and combination therapy with antibacterial agents may be appropriate, given the frequent occurrence of bacterial co‐infection [87]. However, correlations between in vitro susceptibility results and clinical outcomes remain poorly defined due to the limited number of reported invasive cases [88]. The overall reported mortality rate is approximately 50% [87]. However, according to limited reports, the mortality attributable specifically to *A. mycotoxinivorans* remains unclear and further studies are warranted.

Clinical characteristics, risk factors and antifungal susceptibility of rare yeasts were shown in Table 1.

**TABLE 1:** Summary of species misidentification, initial diagnostic methods, risk factors, clinical manifestations, antifungal susceptibility patterns and preferred treatment options for emerging uncommon yeasts reported predominantly in Asia.

Species	Misidentification (initial diagnostic methods)	Risk factors	Clinical manifestations	Antifungal susceptibility patterns based on MIC testing	Preferred treatment options	References
*Candidozyma* (*Candida*) *auris*	*Candida haemulonii* (morphological identification, conventional biochemical test, commercial automated biochemical yeast identification systems, MALDI‐TOF MS) *Candida duobushaemulonii* (morphological identification, commercial automated biochemical yeast identification systems) *Candida lusitaniae* (commercial automated biochemical yeast identification systems, MALDI‐TOF MS)	Advanced age, prior skin colonization, recent surgery, indwelling devices, immunocompromised states (neutropenia, glucocorticoid use, organ transplant recipient)	Skin colonization (nares, axilla, groin), candidaemia, myopericarditis, meningitis, hepatosplenic infections, osteomyelitis, urinary tract infections, endophthalmitis, ear infections, wound infections	Clade‐dependent susceptibility: Azoles: mostly resistance Amphotericin B: variable Echinocandins: mostly susceptible	Echinocandins (especially micafungin), amphotericin B	[22, 23, 24, 92]
*Candidozyma* (*Candida*) *vulturna*	*Candida haemulonii* (commercial biochemical testing, commercial automated biochemical yeast identification systems, MALDI‐TOF MS) *Candida duobushaemulonii* (MALDI‐TOF MS) *Candida pseudohaemulonii* (MALDI‐TOF MS)	Male, immunocompromised states (neutropenia, endovascular catheters, haematologic malignancy)	Catheter‐related blood stream infection	MIC_50_ Fluconazole (*n* = 52): 32 μg/mL Voriconazole (*n* = 52): 0.25 μg/mL Amphotericin B (*n* = 44): 4 μg/mL Micafungin (*n* = 47): 0.06 μg/mL	Echinocandins, amphotericin B	[41, 93]
*Kluyveromyces marxianus* (*Candida kefyr*)	No report	Haematologic malignancies, solid organ (kidney) and haematologic stem cell transplant recipients	Candidaemia, catheter‐related blood stream infection, keratitis	Median MIC Fluconazole (*n* = 6): 0.25 μg/mL Voriconazole (*n* = 4): 0.012 μg/mL Amphotericin B (*n* = 4): 0.5 μg/mL Caspofungin (*n* = 2): 0.019 μg/mL	Fluconazole, amphotericin B, caspofungin, nystatin	[48]
*Pichia* (*Candida*) *norvegensis*	No report	HIV infection, malignancies, kidney transplant, peritoneal dialysis	Oropharyngeal candidiasis, peritonitis	MIC_50_ Fluconazole (*n* = 18): 32 μg/mL Itraconazole (*n* = 18): 0.25 μg/mL Voriconazole (*n* = 18): 0.5 μg/mL Amphotericin B (*n* = 18): 0.25 μg/mL Micafungin (*n* = 18): 0.064 μg/mL	Amphotericin B, echinocandins	[58]
*Candida khanbai*	*Candida duobushaemulonii* (commercial automated biochemical yeast identification systems) *Candida pseudobushaemulonii* (commercial biochemical testing, MALDI‐TOF MS) *Clostridium cadaveris* (MALDI‐TOF MS)	Hospital‐acquired pneumonia	Candidaemia	Median MIC Fluconazole (*n* = 2): > 48 μg/mL Itraconazole (*n* = 2): 12 μg/mL Voriconazole (*n* = 2): 9 μg/mL Amphotericin B (*n* = 2): 4 μg/mL Micafungin (*n* = 2): 0.3125 μg/mL	Echinocandins	[64]
*Lodderomyces elongisporus*	*Candida parapsilosis, Candida stellatoidea, Geotrichum* spp. (commercial biochemical testing)	Catheter‐related infections, neonatal ICU, nosocomial outbreaks	Fungaemia, endocarditis	Median MIC Fluconazole (*n* = 11): 0.25 μg/mL Voriconazole (*n* = 11): 0.015 μg/mL Amphotericin B (*n* = 10): 0.25 μg/mL Micafungin (*n* = 8): 0.032 μg/mL	Echinocandins	[79, 82]
*Apiotrichum mycotoxinivorans*	No report	Catheter‐related infections, post‐major surgery	Fungaemia, complicated intra‐abdominal infection, post‐neurosurgical meningitis	Median MIC Voriconazole (*n* = 2): 0.12 μg/mL Amphotericin B (*n* = 3): 2 μg/mL	Voriconazole	[87]

Abbreviations: HIV, human immunodeficiency virus; ICU, intensive care unit; MALDI‐TOF MS, Matrix‐Assisted Laser Desorption/Ionization Time‐of‐Flight Mass Spectrometry; MIC, minimum inhibitory concentration; *n*, number of isolates tested.

### 3.2 Rare Moulds

#### 3.2.1 Emerging Species of Mucorales

The Mucorales order, a group of non‐septate hyphal fungi that includes *Rhizopus*, *Rhizomucor*, *Mucor*, *Lichtheimia*, *Apophysomyces*, *Cunninghamella* and *Saksenaea*, causes mucormycosis through angioinvasion [94]. The primary route of infection is inhalation of fungal conidia. Typically, mucormycosis presents as rhinocerebral disease in patients with uncontrolled diabetes mellitus and as pulmonary disease in immunocompromised individuals, particularly those with haematologic malignancies undergoing intensive chemotherapy or haematopoietic stem cell transplantation [95]. Among the Mucorales, *Rhizopus* spp., especially *R. arrhizus*, are the most common aetiologic agents of invasive disease. *Rhizopus homothallicus*, previously considered an environmental species, has emerged in over the past decade as a human pathogen, with an increasing number of cases reported in India [96, 97]. A retrospective study from India found that *R. homothallicus* accounted for 6.8% (43/631) of mucormycosis cases [98]. Molecular analysis using 18S rRNA gene sequencing and amplified fragment length polymorphism (AFLP) revealed distinct clustering of *R. homothallicus*, confirming its differentiation from other *Rhizopus* species [98]. Compared to *R. arrhizus*, *R. homothallicus* infections occurred in younger patients (mean age 45.9 vs. 52.5 years), exhibited slower‐growing colonies that were cottony white turning greyish to olive‐brown, with less prominent rhizoids and abundant golden zygospores [98]. The clinical manifestations of *R. homothallicus* were not different from those of *R. arrhizus* infection, except for a significantly higher frequency of visual disturbances (43.9% vs. 12.7%) [98]. Clinically, infections were also associated with a better survival rate (9.8% vs. 3.9%) [98]. Antifungal susceptibility testing demonstrated generally lower MICs for amphotericin B (0.03–16 μg/mL), itraconazole (0.03–16 μg/mL), posaconazole (0.03–8 μg/mL) and isavuconazole (0.03–16 μg/mL) compared to *R. arrhizus* [98]. In addition to *Rhizopus* species, several less common Mucorales have also been identified as human pathogens [99]. *Apophysomyces variabilis*, the second most commonly isolated species in India [99], is associated with necrotizing cutaneous infections in immunocompetent hosts following trauma [100], while *Saksenaea vasiformis* typically causes localized infections after contaminated injuries [101]. *Mucor irregularis* is linked to primary cutaneous disease in immunocompetent individuals [102], and *Thamnostylum lucknowense* has been reported as a rare cause of rhinocerebral mucormycosis in patients with poorly controlled diabetes mellitus [103]. Because conventional morphological identification may be unreliable for rare Mucorales, molecular methods are increasingly important for accurate species identification, epidemiological surveillance and understanding regional diversity.

#### 3.2.2 Filamentous Basidiomycetes

The phylum *Basidiomycota*, which includes fungi commonly referred to as mushrooms, also contains well‐known human pathogenic yeast species such as *Cryptococcus* spp., *Trichosporon* spp. and *Malassezia* spp. Although filamentous basidiomycetes are rare causes of invasive fungal infections in humans, their clinical significance has become increasingly recognized, particularly in the context of chemotherapy, organ transplantation and novel immunosuppressive therapies [16]. Improved post‐transplant outcomes allow recipients to travel more often, increasing exposure to uncommon pathogens. The largest review in 2014 summarized 218 cases worldwide, with *Schizophyllum commune* being the most common species (*n* = 114; 52.3%) [16]. *Schizophyllum commune* is an edible, wood‐decaying fungus that causes respiratory infections, such as sinusitis and allergic bronchopulmonary mycosis (ABPM), and extra‐respiratory disease, including onychomycosis, brain abscess, meningitis and palate ulceration. *Hormographiella aspergillata* (*n* = 13; 5.9%), the anamorph of *Coprinus cinereus* found in compost and sewage, has been linked to invasive infections in patients with haematologic malignancies, including disseminated disease, endophthalmitis, infective endocarditis and pneumonia. *Ceriporia lacerata* (*n* = 11; 5%), a wood‐decaying fungus, was associated with pneumonia in chronic lung disease. A 2024 series described seven *Volvariella volvacea* infections (7/8 from Asia) in transplant or chemotherapy patients, presenting with pneumonia, brain lesions, skin lesions and infective endocarditis [104]. Other reported species include *Inonotus tropicalis*, *Irpex lacteus*, *Phellinus undulatus*, *Perenniporia* spp., *Bjerkandera adusta*, *Sporotrichum pruinosum*, *Phanerochaete sordida* and *Cyclomyces tabacinus*. Most cases occurred in Japan (*n* = 94; 43%) and India (*n* = 57; 26%), with the respiratory tract being the most affected site (*n* = 71), including 34 ABPM cases [16]. Colonies are typically cottony and white. Clamp connections, hyphal bridges that facilitate dikaryon formation, are infrequently observed in clinical isolates, which are generally monokaryotic (*n*); however, dikaryotization (2*n*) can be induced through mating with a compatible strain. Owing to the frequent absence of distinctive morphological features, phenotypic identification is often unreliable, rendering molecular identification essential. This is commonly achieved by sequencing the ribosomal ITS region and the D1/D2 domains of the large subunit (LSU) ribosomal DNA [16]. Antifungal susceptibility testing generally demonstrates low MICs for itraconazole, voriconazole, posaconazole and amphotericin B, whereas MICs for fluconazole and echinocandins are typically elevated. Clinical outcomes have been heterogeneous, and a standardized treatment regimen has yet to be established [16, 104]. Current management relies largely on extrapolation from case reports and expert opinion rather than high‐quality clinical evidence. Reported outcomes are highly heterogeneous and likely influenced by the infecting species, host immune status, disease extent and the feasibility of surgical intervention in addition to antifungal therapy.

#### 3.2.3 Human Non‐*marneffei Talaromyces*

*Talaromyces marneffei* is a thermally dimorphic fungus endemic to southern China and the northern regions of Southeast Asia, including Thailand, with bamboo rats (*Rhizomys* spp.) serving as its natural reservoir [9, 105]. Infection occurs through inhalation of airborne conidia, most often causing systemic fungal disease in individuals with HIV infection [9]. Clinically, it frequently presents with generalized umbilicated papular skin lesions [9]. However, emerging data have identified new risk groups beyond HIV, including patients undergoing cancer treatment, organ transplant recipients and individuals receiving immunosuppressive therapy [9, 106]. Standard treatment includes amphotericin B followed by itraconazole [107]. Between 2006 and 2020 [108, 109, 110, 111, 112], several cases of human talaromycosis caused by non‐*marneffei Talaromyces* species have been reported worldwide, particularly in Asia. Reported species include *T. amestolkiae* [109], *T. purpurogenus* [108], *T. piceus* [110], *T. subaurantiacus* [111], *T. kabodanensis* [111], *T. minnesotensis* [111], *T. rapidus* [111], *T. alveolaris* [111] and *T. atroroseus* [112]. Reported risk factors include HIV infection, haematologic malignancies [108, 109] and chronic granulomatous disease [110]. Pulmonary involvement is the most common clinical manifestation. A notable study from Indonesia examined 10 clinical and environmental isolates of *Talaromyces* spp. recovered from patients and from rats captured in patients' homes [112]. Initially, all isolates were phenotypically identified as *T. marneffei*; however, subsequent molecular analysis revealed that nine of them were actually *T. atroroseus* [112]. These misidentified isolates differed morphologically from *T. marneffei*, lacking soluble red pigment production in agar and showing more filamentous growth, with longer and fragmented hyphae [112]. Antifungal susceptibility testing using Sensititre revealed that *T. atroroseus* exhibits high MICs for amphotericin B and echinocandins but low MICs for itraconazole and posaconazole [112]. As a result, treatment of non‐*marneffei* talaromycosis, particularly due to *T. atroroseus*, should be guided by antifungal susceptibility testing rather than standard protocols alone [112]. Overall, the available evidence indicates that non‐*marneffei Talaromyces* species represent an emerging but poorly characterized group of opportunistic pathogens. Current knowledge is constrained by the rarity of reported cases, publication bias, and reliance on retrospective case reports.

#### 3.2.4 *Emergomyces* spp.

Emergomycosis is a systemic mycosis caused by thermally dimorphic fungi of the genus *Emergomyces*, previously classified within *Emmonsia* and belonging to the phylum Ascomycota [113]. Human infection is presumed to occur through inhalation of airborne conidia originating from soil, where these organisms persist as saprobes [114]. Among recognized species, *Emergomyces africanus* accounts for the majority of cases (71.4%), followed by *E*. *pasteurianus*, *E. canadensis* , *E*. *crescens*, *E. orientalis* and *E. europaeus* [114]. Although more than 70% of reported cases originate from South Africa [115, 116], emergomycosis is increasingly recognized in Asia and North America (each 9.1%). In Asia, infections are predominantly caused by *E*. *pasteurianus* and *E. orientalis* , with cases reported from China, Hong Kong and India [117, 118]. The disease primarily affects immunocompromised individuals, particularly those with advanced HIV infection (CD4 < 100 cells/μL) often accompanied by anaemia or thrombocytopenia, in this population, *E. africanus* is the most frequently implicated species [114]. In contrast, non‐HIV‐infected patients, including solid organ transplant recipients and those with diabetes mellitus or chronic kidney disease, are more commonly infected by non‐*africanus* species [114]. Clinical manifestations range from localized pulmonary disease to disseminated infection and fungaemia. Cutaneous involvement is prominent in HIV‐associated disease, with 75%–95% of patients presenting with polymorphic skin lesions such as papules, nodules, ulcers, verrucous plaques and crusted lesions, whereas non‐HIV patients more often present with predominant respiratory symptoms [114].

Definitive diagnosis relies on a combination of fungal culture and histopathological examination, although findings are frequently nonspecific and may mimic histoplasmosis or blastomycosis [119]. Histopathology typically demonstrates small intracellular yeasts with narrow‐based budding within granulomatous or suppurative dermal infiltrates [118]. Culture remains essential for species‐level identification. On Sabouraud dextrose agar, colonies are yellowish white to tan, initially glabrous, later becoming powdery and furrowed and typically reaching 2.5–3.5 cm in diameter within 3 weeks. At 24°C, the mould phase is characterized by slender conidiophores arising at right angles from hyphae and forming characteristic ‘floret’ structures composed of short secondary conidiophores bearing single, small, subspherical conidia [118]. At 37°C, the yeast phase consists of abundant ovoid yeast cells exhibiting unipolar narrow‐based budding with minimal hyphal elements, confirming thermal dimorphism [118]. Despite antifungal therapy, emergomycosis is associated with high mortality rates ranging from 31% to 46% [118]. Nevertheless, induction therapy with amphotericin B followed by oral itraconazole has been associated with improved clinical outcomes and survival [118]. Emergomycosis is an increasingly recognized opportunistic fungal infection whose epidemiology appears to vary substantially by geographic region, infecting species and host immune status. However, current understanding remains constrained by limited surveillance, diagnostic challenges and reliance on retrospective case reports and observational studies.

#### 3.2.5 *Trichophyton indotineae*

*Trichophyton indotineae*, formerly designated *T. mentagrophytes* genotype VIII, is an emerging anthropophilic dermatophyte first described in India in 2020 [120]. It has since become a significant public health concern owing to its high transmissibility, which occurs predominantly through direct skin‐to‐skin contact. Indirect transmission has also been documented via contaminated fomites and environments, including bathrooms, clothing and bed linens [121]. Clinically, *T. indotineae* most commonly causes tinea corporis, tinea cruris and tinea genitalis, with a rising incidence of tinea faciei [120, 121, 122]. Infections caused by this species are typically more extensive and severe than those associated with other prevalent dermatophytes, such as *T. rubrum* and *T*. *mentagrophytes* [120, 121, 122]. The disease course is frequently chronic and relapsing, characterized by highly inflammatory, scaly, hyperpigmented and intensely pruritic lesions, most often involving the lower trunk and inguinal regions [120, 121, 122]. Since its initial identification, *T*. *indotineae* has been reported across Asia, the Middle East, Europe and the Americas, indicating rapid global dissemination [123]. Morphologically, it is indistinguishable from members of the *T*. *mentagrophytes* complex based on culture characteristics alone [123]. However, biochemical testing may aid preliminary differentiation, as *T*. *indotineae* is urease‐negative, in contrast to *T*. *interdigitale* and *T*. *mentagrophytes*, which are urease‐positive [123]. A major therapeutic challenge is the high prevalence of terbinafine resistance [121]. This resistance is mediated by multiple mechanisms, including efflux pump overexpression, biofilm formation and point mutations in the ERG1 gene encoding squalene epoxidase (SQLE), which reduce antifungal binding affinity [121].

Systemic itraconazole remains the treatment of choice. Standard dosing of 200 mg daily for 2 weeks, with extension up to 12 weeks in refractory cases, has demonstrated favourable outcomes, with no clear benefit observed from higher dosing regimens [121]. The super‐bioavailable (SUBA)‐itraconazole formulation, administered at 50 mg twice daily for 4 weeks, has also shown good clinical efficacy [121]. Adjunctive topical therapy with azoles, including clotrimazole, ketoconazole, luliconazole or bifonazole, may further improve treatment response when combined with systemic antifungal therapy [124]. Topical agents alone are generally insufficient for disease eradication but may serve a supportive role [121]. Although clinical experience remains limited, voriconazole has demonstrated promising in vitro activity and may be considered as salvage therapy in refractory infections [121]. In contrast, other systemic antifungal agents, including terbinafine, fluconazole, ketoconazole and griseofulvin, generally exhibit limited clinical efficacy against *T*. *indotineae* [121]. *Trichophyton indotineae* represents an important example of the intersection between antifungal resistance, inappropriate antifungal prescribing practices, and global dissemination. While evidence supporting its emergence and resistance is compelling, current management strategies rely largely on observational data from South Asia.

We summarized the risk factors, clinical manifestations, antifungal susceptibility patterns and treatment options for emerging uncommon moulds in Asia in Table 2.

**TABLE 2:** Summary of risk factors, clinical manifestations, antifungal susceptibility patterns and preferred treatment options for emerging uncommon moulds reported predominantly in Asia.

Species	Risk factors	Clinical manifestations	Antifungal susceptibility patterns based on MIC testing	Preferred treatment options	References
*Rhizopus homothallicus*	Diabetic ketoacidosis	Pulmonary mucormycosis, cutaneous mucormycosis, rhinocerebral mucormycosis	MIC range Itraconazole (*n* = 34): 0.03–16 μg/mL Posaconazole (*n* = 34): 0.03–8 μg/mL Isavuconazole (*n* = 34): 0.03–16 μg/mL Amphotericin B (*n* = 34): 0.03–16 μg/mL Terbinafine (*n* = 34): 0.03–16 μg/mL	Amphotericin B, posaconazole	[98]
*Schizophyllum commune*	Normal host	Sinusitis, allergic bronchopulmonary mycosis, brain abscess	MIC range Itraconazole (*n* = 2): < 0.06–0.125 μg/mL Posaconazole (*n* = 2): 0.015–0.125 μg/mL Isavuconazole (*n* = 2): 0.125–0.25 μg/mL Amphotericin B (*n* = 2): 0.5–1.0 μg/mL MEC range Echinocandins (*n* = 2): 0.125–0.5 μg/mL	Itraconazole, voriconazole, posaconazole, amphotericin B	[125, 126]
*Hormographiella aspergillata*	Haematologic malignancies	Disseminated disease, endophthalmitis, infective endocarditis, pneumonia	MIC range Itraconazole (*n* = 11): 0.25 to ≥ 32 μg/mL Voriconazole (*n* = 9): 0.015–1 μg/mL Posaconazole (*n* = 6): 0.06–2 μg/mL Amphotericin B (*n* = 16): 0.03–32 μg/mL Micafungin (*n* = 9): 0.25 to ≥ 16 μg/mL	Voriconazole, amphotericin B	[127]
*Volvariella volvacea*	Haematologic malignancies, organ transplant recipient	Brain abscess, pulmonary infection, skin infection, infective endocarditis	MIC range Itraconazole (*n* = 2): 0.12–0.5 μg/mL Voriconazole (*n* = 2): 0.5–2.0 μg/mL Posaconazole (*n* = 2): 0.5–2.0 μg/mL Amphotericin B (*n* = 2): 0.5–2.0 μg/mL Echinocandins (*n* = 2): > 8 μg/mL	Voriconazole, amphotericin B	[128, 129]
Non‐*marneffei Talaromyces*	HIV infection, haematologic malignancies, chronic granulomatous disease	Pulmonary infection	MIC range for *T. atroroseus*: Itraconazole (*n* = 5): 0.12 to > 16 μg/mL Voriconazole (*n* = 5): 0.12 to > 8 μg/mL Posaconazole (*n* = 5): 0.25 to > 8 μg/mL Amphotericin B (*n* = 5): 0.5 to > 8 μg/mL Micafungin (*n* = 2): 0.12 to > 8 μg/mL	Should be based on susceptibility testing	[112]
*Emergomyces spasteurianus*	HIV infection, solid organ transplant recipients, diabetes mellitus, chronic kidney disease	Pulmonary infection, cutaneous infection, disseminated infection	MIC range for *E. pasteurianus*: Itraconazole (*n* = 5): 0.063–0.25 μg/mL Voriconazole (*n* = 5): 0.25 μg/mL Posaconazole (*n* = 5): 0.063–0.125 μg/mL Amphotericin B (*n* = 5): 0.031–0.125 μg/mL Micafungin (*n* = 2): 0.016–0.063 μg/mL	Amphotericin B induction followed by oral itraconazole	[130]
*Trichophyton indotineae*	Immunocompetent host	Severe dermatophytosis	MIC range Itraconazole (*n* = 16): 0.032–2 μg/mL Voriconazole (*n* = 5): 0.032–1 μg/mL Posaconazole (*n* = 5): 0.063–0.5 μg/mL Terbinafine (*n* = 16): 0.002 to ≥ 128 μg/mL Amphotericin B (*n* = 5): 2–4 μg/mL Micafungin (*n* = 2): < 0.001–0.032 μg/mL	Systemic itraconazole	[131]

Abbreviations: HIV, human immunodeficiency virus; MEC, minimal effective concentration; MIC, minimum inhibitory concentrations; *n*, number of isolates tested.

### 3.3 Pseudo‐Fungi

#### 3.3.1 Oomycetes

Oomycetes are fungal‐like organisms classified within the class Oomycota. Two genera, *Pythium* and *Lagenidium*, are recognized causes of human infection [132]. Species of *Pythium*, most notably *P. insidiosum*, *P. periculosum* and *P. aphanidermatum* , are responsible for pythiosis [133], whereas lagenidiosis is caused primarily by *L. giganteum* and *L*. *deciduum* [134]. Pythiosis predominantly occurs in tropical and subtropical regions, including Thailand, Brazil and parts of the United States, and is typically associated with exposure to contaminated freshwater environments [135]. The disease manifests in several clinical forms. Vascular pythiosis represents the most severe presentation and is frequently observed in patients with underlying haematologic disorders, such as haemoglobinopathies or paroxysmal nocturnal haemoglobinuria (PNH) [136, 137]; ocular involvement, including keratitis and exogenous endophthalmitis, is more commonly reported in immunocompetent individuals [138, 139]. Cutaneous and subcutaneous pythiosis typically presents as chronic ulcerative lesions [140]. Diagnosis is often challenging because conventional cultures reveal hyaline, broad, non‐septate hyphae that closely resemble those seen in mucormycosis [141]. Ancillary diagnostic methods, including zoospore induction, molecular assays and serological testing, are essential for definitive identification [141, 142]. Surgical resection remains the cornerstone of management, with improved outcomes observed when combined with adjunctive antimicrobial therapy, such as azithromycin, doxycycline and itraconazole [143]. A study from Thailand demonstrated that residual disease following surgery was associated with significantly higher mortality compared with complete resection (31% vs. 15.7%), underscoring the critical importance of aggressive surgical management [143]. Serial monitoring of serum β‐d‐glucan levels may also be useful in assessing disease activity and treatment response [143].

Lagenidiosis is exceedingly rare in humans. First described in animals in the United States in 2003 [144], only a limited number of human cases have been reported, including cutaneous infection in a patient with acute myeloid leukaemia following haematopoietic stem cell transplantation after freshwater exposure [145] and a keratitis [146]. In culture, *Lagenidium* species grow rapidly on Sabouraud dextrose agar at 37°C, producing cream‐coloured, glabrous colonies [146]. Microscopic examination reveals broad, non‐septate hyphae with right‐angle branching, occasionally exhibiting coiling or vacuolation [146]. Zoospore induction can be achieved using boiled grass blades [142]. As with pythiosis, lagenidiosis may be misdiagnosed as mucormycosis based on histopathological features alone [146]. Definitive diagnosis requires molecular identification, typically through sequencing of the ITS and cytochrome oxidase subunit II (COXII) regions. Surgical intervention remains the mainstay of therapy, while antifungal options are limited; caspofungin and terbinafine have shown some activity, whereas azoles are generally ineffective due to the minimal ergosterol content of the oomycete cell membrane [146]. Much of the current evidence of human pythiosis and lagenidiosis originates from endemic countries, particularly Thailand, and consists primarily of retrospective cohorts and individual case reports. Improved clinician awareness and wider availability of molecular diagnostics will be essential to better define the epidemiology, optimize management, and improve outcomes.

### 3.4 Challenges in Antifungal Susceptibility Testing

Antifungal susceptibility testing has become increasingly important with the emergence of rare fungal pathogens exhibiting diverse intrinsic and acquired resistance mechanisms. However, antifungal susceptibility testing is evolving and translating in vitro susceptibility results into clinical decision‐making remains challenging. Although standardized antifungal susceptibility testing methodologies have been established in certain fungal pathogens, species‐specific clinical breakpoints are available for only a limited number of common yeasts and moulds [147] Consequently, for many emerging pathogens in this review, MIC values are unable to accurately categorize as susceptible, intermediate or resistant. Furthermore, epidemiological cutoff values (ECVs/ECOFFs) are lacking for most of these emerging organisms, making it difficult to distinguish wild‐type isolates from those harbouring acquired resistance mechanisms. Additional challenges arise from methodological differences, limited reproducibility for certain mould species, and the uncertain correlation between MIC values and clinical outcomes [148]. Some rare fungi also exhibit paradoxical growth, trailing effects or poor sporulation, further complicating susceptibility testing and interpretation [147]. As a result, antifungal susceptibility testing should not be interpreted in isolation but rather integrated with accurate species identification and clinical information.

These limitations highlight the urgent need for regional and global surveillance initiatives. Asia has emerged as a hotspot for several rare fungal pathogens, yet susceptibility data remain fragmented and are often derived from small, single‐centre studies. Large multicentre epidemiological studies are needed to define species distribution, establish regional MIC distributions, characterize emerging resistance mechanisms and correlate antifungal susceptibility testing with clinical outcomes. Strengthening laboratory capacity and establishing collaborative surveillance networks across Asia should therefore be considered a priority to improve the management of emerging rare fungal infections.

## 4 Conclusions

Rare fungi and fungal‐like pathogens represent an under‐recognized but clinically significant threat, particularly in resource‐limited settings. Their identification remains challenging due to nonspecific clinical presentations and morphological similarities with more common fungi, often necessitating advanced molecular techniques such as sequencing for definitive diagnosis. However, suboptimal laboratory infrastructure in many parts of Asia limits access to these tools, contributing to delays in accurate identification. Such delays can facilitate ongoing transmission, increase the risk of outbreaks and result in a higher burden of disease. Furthermore, the lack of established antifungal susceptibility breakpoints for many of these organisms complicates therapeutic decision‐making, frequently leading to suboptimal treatment. Collectively, these factors contribute to the persistently high mortality associated with infections caused by rare yeasts and moulds, underscoring the urgent need to strengthen diagnostic capacity, improve surveillance and develop standardized treatment guidelines.

## Author Contributions

**Narut Chancharussin:** writing – review and editing, writing – original draft, methodology, conceptualization, data curation, formal analysis. **Anupop Jitmuang:** writing – review and editing, conceptualization, methodology, writing – original draft, validation, formal analysis. **Arunaloke Chakrabarti:** writing – review and editing, methodology, conceptualization, formal analysis. **Methee Chayakulkeeree:** writing – review and editing, conceptualization, supervision, methodology, validation, writing – original draft, formal analysis.

## Funding

The authors have nothing to report.

## Ethics Statement

The authors confirm that the ethical policies of the journal, as noted on the journal's author guidelines page, have been adhered to. No ethical approval was required as this is a review article with no original research data.

## Conflicts of Interest

The authors declare no conflicts of interest.

## Data Availability

Data sharing not applicable to this article as no datasets were generated or analysed during the current study.

## References

[1] N. van Rhijn and M. Bromley , “The Consequences of Our Changing Environment on Life Threatening and Debilitating Fungal Diseases in Humans,” Journal of Fungi 7, no. 5 (2021): 367. 34067211 10.3390/jof7050367 PMC8151111

[2] M. E. George , T. T. Gaitor , D. B. Cluck , A. F. Henao‐Martínez , N. R. Sells , and D. B. Chastain , “The Impact of Climate Change on the Epidemiology of Fungal Infections: Implications for Diagnosis, Treatment, and Public Health Strategies,” Therapeutic Advances in Infectious Disease 12 (2025): 20499361251313841. 39944519 10.1177/20499361251313841 PMC11815821

[3] M. A. Slavin and A. Chakrabarti , “Opportunistic Fungal Infections in the Asia‐Pacific Region,” Medical Mycology 50, no. 1 (2012): 18–25. 21905945 10.3109/13693786.2011.602989

[4] S. R. Lockhart , A. Chowdhary , and J. A. W. Gold , “The Rapid Emergence of Antifungal‐Resistant Human‐Pathogenic Fungi,” Nature Reviews. Microbiology 21, no. 12 (2023): 818–832. 37648790 10.1038/s41579-023-00960-9 PMC10859884

[5] D. Seidel , S. Wurster , J. D. Jenks , et al., “Impact of Climate Change and Natural Disasters on Fungal Infections,” Lancet Microbe 5, no. 6 (2024): e594–e605. 38518791 10.1016/S2666-5247(24)00039-9

[6] A. Chakrabarti and M. A. Slavin , “Endemic Fungal Infections in the Asia‐Pacific Region,” Medical Mycology 49, no. 4 (2011): 337–344. 21254966 10.3109/13693786.2010.551426

[7] H. Prakash and A. Chakrabarti , “Global Epidemiology of Mucormycosis,” Journal of Fungi 5, no. 1 (2019): 26. 30901907 10.3390/jof5010026 PMC6462913

[8] Y. Qin , X. Huang , H. Chen , et al., “Burden of *Talaromyces marneffei* Infection in People Living With HIV/AIDS in Asia During ART Era: A Systematic Review and Meta‐Analysis,” BMC Infectious Diseases 20, no. 1 (2020): 551. 32727383 10.1186/s12879-020-05260-8 PMC7392840

[9] F. Wang , R. Han , and S. Chen , “An Overlooked and Underrated Endemic Mycosis ‐ Talaromycosis and the Pathogenic Fungus *Talaromyces marneffei* ,” Clinical Microbiology Reviews 36, no. 1 (2023): e00051‐22. 36648228 10.1128/cmr.00051-22 PMC10035316

[10] O. Bupha‐Intr , C. Butters , G. Reynolds , et al., “Consensus Guidelines for the Diagnosis and Management of Invasive Fungal Disease due to Moulds Other Than *Aspergillus* in the Haematology/Oncology Setting, 2021,” Internal Medicine Journal 51, no. Suppl 7 (2021): 177–219. 34937139 10.1111/imj.15592

[11] M. Hoenigl , J. Salmanton‐García , T. J. Walsh , et al., “Global Guideline for the Diagnosis and Management of Rare Mould Infections: An Initiative of the European Confederation of Medical Mycology in Cooperation With the International Society for Human and Animal Mycology and the American Society for Microbiology,” Lancet Infectious Diseases 21, no. 8 (2021): e246–e257. 33606997 10.1016/S1473-3099(20)30784-2

[12] S. Ahmad , Z. Khan , N. Al‐Sweih , W. Alfouzan , L. Joseph , and M. Asadzadeh , “ *Candida kefyr* in Kuwait: Prevalence, Antifungal Drug Susceptibility and Genotypic Heterogeneity,” PLoS One 15, no. 10 (2020): e0240426. 33108361 10.1371/journal.pone.0240426 PMC7591085

[13] J. Guitard , A. Angoulvant , V. Letscher‐Bru , et al., “Invasive Infections due to *Candida norvegensis* and *Candida inconspicua*: Report of 12 Cases and Review of the Literature,” Medical Mycology 51, no. 8 (2013): 795–799. 23855412 10.3109/13693786.2013.807444

[14] S. Ionescu , I. Luchian , C. Damian , et al., “ *Candida auris* Updates: Outbreak Evaluation Through Molecular Assays and Antifungal Stewardship—A Narrative Review,” Current Issues in Molecular Biology 46, no. 6 (2024): 6069–6084. 38921033 10.3390/cimb46060362 PMC11202268

[15] C. A. Boutin , F. Durocher , S. Beauchemin , D. Ziegler , C. N. Abou Chakra , and S. F. Dufresne , “Breakthrough Invasive Fungal Infections in Patients With High‐Risk Hematological Disorders Receiving Voriconazole and Posaconazole Prophylaxis: A Systematic Review,” Clinical Infectious Diseases 79, no. 1 (2024): 151–160. 38752732 10.1093/cid/ciae203 PMC11259221

[16] A. Chowdhary , S. Kathuria , K. Agarwal , and J. F. Meis , “Recognizing Filamentous Basidiomycetes as Agents of Human Disease: A Review,” Medical Mycology 52, no. 8 (2014): 782–797. 25202126 10.1093/mmy/myu047

[17] L. Liao , K. Pan , D. Zheng , S. Wang , W. Wu , and C. Cao , “Exploring the Diversity and Pathogenicity of *Talaromyces* Species Isolated From Clinical in Southern China,” Frontiers in Microbiology 16 (2025): 1610481. 40641883 10.3389/fmicb.2025.1610481 PMC12241079

[18] B. H. Tan , A. Chakrabarti , A. Patel , et al., “Clinicians' Challenges in Managing Patients With Invasive Fungal Diseases in Seven Asian Countries: An Asia Fungal Working Group (AFWG) Survey,” International Journal of Infectious Diseases 95 (2020): 471–480. 31945491 10.1016/j.ijid.2020.01.007

[19] N. van Rhijn and P. L. White , “Antifungal Treatment Strategies and Their Impact on Resistance Development in Clinical Settings,” Journal of Antimicrobial Chemotherapy 80, no. 12 (2025): 3208–3226. 41092294 10.1093/jac/dkaf382 PMC12670168

[20] J. Geddes‐McAlister and R. S. Shapiro , “New Pathogens, New Tricks: Emerging, Drug‐Resistant Fungal Pathogens and Future Prospects for Antifungal Therapeutics,” Annals of the New York Academy of Sciences 1435, no. 1 (2019): 57–78. 29762860 10.1111/nyas.13739

[21] K. Satoh , K. Makimura , Y. Hasumi , Y. Nishiyama , K. Uchida , and H. Yamaguchi , “ *Candida auris* sp. nov., a Novel Ascomycetous Yeast Isolated From the External Ear Canal of an Inpatient in a Japanese Hospital,” Microbiology and Immunology 53, no. 1 (2009): 41–44. 19161556 10.1111/j.1348-0421.2008.00083.x

[22] T. Khan , N. I. Faysal , M. M. Hossain , et al., “Emergence of the Novel Sixth *Candida auris* Clade VI in Bangladesh,” Microbiology Spectrum 12, no. 7 (2024): e03540‐23. 38842332 10.1128/spectrum.03540-23 PMC11218448

[23] N. A. Chow , J. F. Muñoz , L. Gade , et al., “Tracing the Evolutionary History and Global Expansion of *Candida auris* Using Population Genomic Analyses,” MBio 11, no. 2 (2020): e03364‐19. 32345637 10.1128/mBio.03364-19 PMC7188998

[24] C. Suphavilai , K. K. K. Ko , K. M. Lim , et al., “Detection and Characterisation of a Sixth *Candida auris* Clade in Singapore: A Genomic and Phenotypic Study,” Lancet Microbe 5, no. 9 (2024): 100878. 39008997 10.1016/S2666-5247(24)00101-0

[25] V. M. Horton and J. E. Nett , “ *Candida auris* Infection and Biofilm Formation: Going Beyond the Surface,” Current Clinical Microbiology Reports 7, no. 3 (2020): 51–56. 33178552 10.1007/s40588-020-00143-7 PMC7654955

[26] A. Ware , W. Johnston , C. Delaney , et al., “Dry Surface Biofilm Formation by *Candida auris* Facilitates Persistence and Tolerance to Sodium Hypochlorite,” APMIS 133, no. 4 (2025): e70022. 40194790 10.1111/apm.70022 PMC11975465

[27] M. Gómez‐Gaviria , J. A. Martínez‐Álvarez , J. O. Chávez‐Santiago , and H. M. Mora‐Montes , “ *Candida haemulonii* Complex and *Candida auris*: Biology, Virulence Factors, Immune Response, and Multidrug Resistance,” Infection and Drug Resistance 16 (2023): 1455–1470. 36942024 10.2147/IDR.S402754 PMC10024503

[28] G. Bravo Ruiz , Z. K. Ross , N. A. R. Gow , and A. Lorenz , “Pseudohyphal Growth of the Emerging Pathogen *Candida auris* Is Triggered by Genotoxic Stress Through the S Phase Checkpoint,” mSphere 5, no. 2 (2020): e00151‐20. 32161147 10.1128/mSphere.00151-20 PMC7067593

[29] L. M. Bentz , N. Le , B. Min , et al., “Evaluation of CHROMagar Candida Plus for the Detection of *C. auris* With a Panel of 206 Fungal Isolates and 83 Colonization Screening Skin‐Swabs,” Microbiology Spectrum 12, no. 4 (2024): e03564‐23. 38364098 10.1128/spectrum.03564-23 PMC10986463

[30] D. J. Santana , J. A. E. Anku , G. Zhao , et al., “A *Candida auris*‐Specific Adhesin, Scf1, Governs Surface Association, Colonization, and Virulence,” Science 381, no. 6665 (2023): 1461–1467. 37769084 10.1126/science.adf8972 PMC11235122

[31] T. de Groot , A. Chowdhary , J. F. Meis , and A. Voss , “Killing of *Candida auris* by UV‐C: Importance of Exposure Time and Distance,” Mycoses 62, no. 5 (2019): 408–412. 30748018 10.1111/myc.12903 PMC6850319

[32] R. AlJindan , D. M. AlEraky , N. Mahmoud , et al., “Drug Resistance‐Associated Mutations in ERG11 of Multidrug‐Resistant *Candida auris* in a Tertiary Care Hospital of Eastern Saudi Arabia,” Journal of Fungi 7, no. 1 (2021): 18. 10.3390/jof7010018 PMC7824384 33396402

[33] J. Li , A. T. Coste , M. Liechti , D. Bachmann , D. Sanglard , and F. Lamoth , “Novel *ERG11* and *TAC1b* Mutations Associated With Azole Resistance in *Candida auris* ,” Antimicrobial Agents and Chemotherapy 65, no. 5 (2021): e02663‐20. 33619054 10.1128/AAC.02663-20 PMC8092887

[34] B. Williamson , A. Wilk , K. D. Guerrero , et al., “Impact of Erg11 Amino Acid Substitutions Identified in *Candida auris* Clade III Isolates on Triazole Drug Susceptibility,” Antimicrobial Agents and Chemotherapy 66, no. 1 (2022): e01624‐21. 34633842 10.1128/AAC.01624-21 PMC8765314

[35] J. Chen , S. Tian , X. Han , et al., “Is the Superbug Fungus Really So Scary? A Systematic Review and Meta‐Analysis of Global Epidemiology and Mortality of *Candida auris* ,” BMC Infectious Diseases 20, no. 1 (2020): 827. 33176724 10.1186/s12879-020-05543-0 PMC7656719

[36] B. Long , A. J. Lacy , A. Koyfman , and S. Y. Liang , “ *Candida auris*: A Focused Review for Emergency Clinicians,” American Journal of Emergency Medicine 84 (2024): 162–167. 39137491 10.1016/j.ajem.2024.07.062

[37] A. Ruiz‐Gaitán , H. Martínez , A. M. Moret , et al., “Detection and Treatment of *Candida auris* in an Outbreak Situation: Risk Factors for Developing Colonization and Candidemia by This New Species in Critically Ill Patients,” Expert Review of Anti‐Infective Therapy 17, no. 4 (2019): 295–305. 30922129 10.1080/14787210.2019.1592675

[38] S. Jaggavarapu , E. M. Burd , and D. S. Weiss , “Micafungin and Amphotericin B Synergy Against *Candida auris* ,” Lancet Microbe 1, no. 8 (2020): e314–e315. 35544183 10.1016/S2666-5247(20)30194-4

[39] Y. Elgammal , E. A. Salama , and M. N. Seleem , “Enhanced Antifungal Activity of Posaconazole Against *Candida auris* by HIV Protease Inhibitors, Atazanavir and Saquinavir,” Scientific Reports 14, no. 1 (2024): 1571. 38238403 10.1038/s41598-024-52012-8 PMC10796399

[40] A. T. de Macedo , D. W. C. L. Santos , B. Spruijtenburg , et al., “Clonal Outbreak of *Candida vulturna* in a Paediatric Oncology Ward in Maranhão, Brazil,” Journal of Infection 89, no. 6 (2024): 106349. 39537034 10.1016/j.jinf.2024.106349

[41] R. Harchand , B. Spruijtenburg , E. F. J. Meijer , T. de Groot , S. M. Rudramurthy , and J. F. Meis , “ *Candida vulturna*, the Next Fungal Menace? A Narrative Review,” Mycoses 68, no. 5 (2025): e70070. 40396413 10.1111/myc.70070 PMC12093444

[42] H. Du , J. Bing , X. Xu , et al., “ *Candida vulturna* Outbreak Caused by Cluster of Multidrug‐Resistant Strains, China,” Emerging Infectious Diseases 29, no. 7 (2023): 1425–1428. 37347816 10.3201/eid2907.230254 PMC10310381

[43] M. Sipiczki and R. M. Tap , “ *Candida vulturna* Pro Tempore sp. nov., a Dimorphic Yeast Species Related to the *Candida haemulonis* Species Complex Isolated From Flowers and Clinical Sample,” International Journal of Systematic and Evolutionary Microbiology 66, no. 10 (2016): 4009–4015. 27411802 10.1099/ijsem.0.001302

[44] A. Muthusamy , M. Rao , A. Chakrabarti , and R. D. Velayuthan , “Case Report: Catheter Related Blood Stream Infection Caused by *Candida vulturna* ,” Medical Mycology Case Reports 36 (2022): 27–30. 35495370 10.1016/j.mmcr.2022.04.001 PMC9043870

[45] Y. B. Oh , H. J. Lim , S. A. Byun , et al., “ *Candida vulturna* Fungemia in an Infant With Congenital Megacolon From The Philippines,” Annals of Laboratory Medicine 43, no. 4 (2023): 395–397. 36843411 10.3343/alm.2023.43.4.395 PMC9989534

[46] R. Mohd Tap , F. Amran , S. Mohamed Sukur , et al., “Epidemiology and Susceptibility Pattern of *Candida vulturna* in Malaysia: A Multidrug Resistance Species and Closely Related to *Candida auris* ,” International Journal of Infectious Diseases 130, no. Suppl 2 (2023): S90.

[47] M. A. Pfaller , D. J. Diekema , J. D. Turnidge , M. Castanheira , and R. N. Jones , “Twenty Years of the SENTRY Antifungal Surveillance Program: Results for *Candida* Species From 1997‐2016,” Open Forum Infectious Diseases 6, no. Suppl 1 (2019): S79–S94. 30895218 10.1093/ofid/ofy358 PMC6419901

[48] Y. Nadir , “ *Candida kefyr* as an Emerging Cause of Invasive Fungal Infection in Transplant Patients: Case Report and Literature Review,” Diagnostic Microbiology and Infectious Disease 113, no. 4 (2025): 117052. 40812262 10.1016/j.diagmicrobio.2025.117052

[49] H. Nouraei , S. Zare , M. Nemati , et al., “Comparative Analysis of Enzymatic Profiles and Biofilm Formation in Clinical and Environmental *Candida kefyr* Isolates,” Environmental Microbiology Reports 16, no. 3 (2024): e13282. 38923398 10.1111/1758-2229.13282 PMC11194042

[50] B. G. Leask and D. Yarrow , “ *Pichia norvegensis* sp. nov,” Sabouraudia 14, no. 1 (1976): 61–63. 1265575

[51] E. Dietrichson , “Étude d'Une Collection Norvégienne de Levures (2e Partie) (Suite et Fin),” Annales de Parasitologie Humaine et Comparée 29, no. 4 (1954): 460–498. 13218481

[52] S. V. Hood , C. B. Moore , and D. W. Denning , “Isolation of *Candida norvegensis* From Clinical Specimens: Four Case Reports,” Clinical Infectious Diseases 23, no. 5 (1996): 1185–1187. 8922831 10.1093/clinids/23.5.1185

[53] N. Kiraz , O. M. Akay , Y. Sen , V. Aslan , Y. Akgun , and Z. Gulbas , “ *Candida norvegensis* Fungaemia in a Neutropenic Patient With Acute Non‐Lymphoblastic Leukaemia,” Mycoses 53, no. 5 (2010): 460–462. 19496937 10.1111/j.1439-0507.2009.01724.x

[54] P. Sandven , K. Nilsen , A. Digranes , T. Tjade , and J. Lassen , “ *Candida norvegensis* : A Fluconazole‐Resistant Species,” Antimicrobial Agents and Chemotherapy 41, no. 6 (1997): 1375–1376. 9174202 10.1128/aac.41.6.1375 PMC163918

[55] H. Nielsen , J. Stenderup , B. Bruun , and J. Ladefoged , “ *Candida norvegensis* Peritonitis and Invasive Disease in a Patient on Continuous Ambulatory Peritoneal Dialysis,” Journal of Clinical Microbiology 28, no. 7 (1990): 1664–1665. 2380389 10.1128/jcm.28.7.1664-1665.1990 PMC268012

[56] M. A. Pfaller , D. J. Diekema , D. L. Gibbs , et al., “Results From the ARTEMIS DISK Global Antifungal Surveillance Study, 1997 to 2007: A 10.5‐Year Analysis of Susceptibilities of *Candida* Species to Fluconazole and Voriconazole as Determined by CLSI Standardized Disk Diffusion,” Journal of Clinical Microbiology 48, no. 4 (2010): 1366–1377. 20164282 10.1128/JCM.02117-09 PMC2849609

[57] S. J. Huang , Y. H. Song , G. Lv , et al., “Emergence of Invasive Candidiasis With Multiple *Candida* Species Exhibiting Azole and Echinocandin Resistance,” Frontiers in Microbiology 16 (2025): 1550894. 40201445 10.3389/fmicb.2025.1550894 PMC11975943

[58] A. A. Stavrou , A. Pérez‐Hansen , M. Lackner , C. Lass‐Flörl , and T. Boekhout , “Elevated Minimum Inhibitory Concentrations to Antifungal Drugs Prevail in 14 Rare Species of Candidemia‐Causing Saccharomycotina Yeasts,” Medical Mycology 58, no. 7 (2020): 987–995. 32043147 10.1093/mmy/myaa005

[59] S. Kumar , A. Kumar , M. Roudbary , R. Mohammadi , L. Černáková , and C. F. Rodrigues , “Overview on the Infections Related to Rare *Candida* Species,” Pathogens 11, no. 9 (2022): 963. 36145394 10.3390/pathogens11090963 PMC9505029

[60] V. Krcmery and A. J. Barnes , “Non‐*albicans Candida* spp. Causing Fungaemia: Pathogenicity and Antifungal Resistance,” Journal of Hospital Infection 50, no. 4 (2002): 243–260. 12014897 10.1053/jhin.2001.1151

[61] S. Arikan , B. Sancak , and G. Hascelik , “ *In Vitro* Activity of Caspofungin Compared to Amphotericin B, Fluconazole, and Itraconazole Against *Candida* Strains Isolated in a Turkish University Hospital,” Medical Mycology 43, no. 2 (2005): 171–178. 15832560 10.1080/13693780410001731565

[62] M. Cuenca‐Estrella , A. Gomez‐Lopez , E. Mellado , A. Monzon , M. J. Buitrago , and J. L. Rodriguez‐Tudela , “Activity Profile *In Vitro* of Micafungin Against Spanish Clinical Isolates of Common and Emerging Species of Yeasts and Molds,” Antimicrobial Agents and Chemotherapy 53, no. 5 (2009): 2192–2195. 19223630 10.1128/AAC.01543-08 PMC2681500

[63] T. Sugita , K. Takeo , M. Ohkusu , et al., “Fluconazole‐Resistant Pathogens *Candida inconspicua* and *C. norvegensis* : DNA Sequence Diversity of the rRNA Intergenic Spacer Region, Antifungal Drug Susceptibility, and Extracellular Enzyme Production,” Microbiology and Immunology 48, no. 10 (2004): 761–766. 15502409 10.1111/j.1348-0421.2004.tb03602.x

[64] A. W. de Jong , K. Al‐Obaid , R. Mohd Tap , et al., “ *Candida khanbhai* sp. nov., a New Clinically Relevant Yeast Within the *Candida haemulonii* Species Complex,” Medical Mycology 61, no. 2 (2023): myad009. 36694950 10.1093/mmy/myad009 PMC9936790

[65] C. R. Arias , J. K. Burns , L. M. Friedrich , R. M. Goodrich , and M. E. Parish , “Yeast Species Associated With Orange Juice: Evaluation of Different Identification Methods,” Applied and Environmental Microbiology 68, no. 4 (2002): 1955–1961. 11916718 10.1128/AEM.68.4.1955-1961.2002 PMC123878

[66] J. P. van der Walt , “ *Lodderomyces*, a New Genus of the Saccharomycetaceae,” Antonie Van Leeuwenhoek 32, no. 1 (1966): 1–5. 5296604 10.1007/BF02097439

[67] A. Adel , A. El‐Baz , Y. Shetaia , and N. M. Sorour , “Biosynthesis of Polyunsaturated Fatty Acids by Two Newly Cold‐Adapted Egyptian Marine Yeast,” 3 Biotech 11, no. 11 (2021): 461. 10.1007/s13205-021-03010-4 PMC8520562 34692369

[68] R. Nualmalang , N. Thanomsridetchai , Y. Teethaisong , P. Sukphopetch , and M. Tangwattanachuleeporn , “Identification of Pathogenic and Opportunistic Yeasts in Pigeon Excreta by MALDI‐TOF Mass Spectrometry and Their Prevalence in Chon Buri Province, Thailand,” International Journal of Environmental Research and Public Health 20, no. 4 (2023): 3191. 36833884 10.3390/ijerph20043191 PMC9967633

[69] S. O. Suh , N. H. Nguyen , and M. Blackwell , “Yeasts Isolated From Plant‐Associated Beetles and Other Insects: Seven Novel *Candida* Species Near *Candida albicans* ,” FEMS Yeast Research 8, no. 1 (2008): 88–102. 17986254 10.1111/j.1567-1364.2007.00320.x

[70] J. Ruiz , N. Ortega , M. Martín‐Santamaría , et al., “Occurrence and Enological Properties of Two New Non‐Conventional Yeasts (*Nakazawaea ishiwadae* and *Lodderomyces elongisporus*) in Wine Fermentations,” International Journal of Food Microbiology 305 (2019): 108255. 31252247 10.1016/j.ijfoodmicro.2019.108255

[71] A. Yadav , P. Jain , K. Jain , et al., “Genomic Analyses of a Fungemia Outbreak Caused by *Lodderomyces elongisporus* in a Neonatal Intensive Care Unit in Delhi, India,” MBio 14, no. 3 (2023): e00636‐23. 37102715 10.1128/mbio.00636-23 PMC10294660

[72] C. M. da Silva , A. M. R. de Carvalho , D. P. C. Macêdo , M. B. Jucá , R. J. M. Amorim , and R. P. Neves , “Candidemia in Brazilian Neonatal Intensive Care Units: Risk Factors, Epidemiology, and Antifungal Resistance,” Brazilian Journal of Microbiology 54, no. 2 (2023): 817–825. 36892755 10.1007/s42770-023-00943-1 PMC10235359

[73] Y. Wang and J. Xu , “ *Lodderomyces elongisporus*: An Emerging Human Fungal Pathogen,” PLoS Pathogens 19, no. 9 (2023): e1011613. 37676851 10.1371/journal.ppat.1011613 PMC10484426

[74] T. Dear , Y. Joe Yu , S. Pandey , J. Fuller , and M. K. Devlin , “The First Described Case of *Lodderomyces elongisporus* Meningitis,” Journal of the Association of Medical Microbiology and Infectious Disease Canada 6, no. 3 (2021): 221–228. 36337753 10.3138/jammi-2021-0006 PMC9615464

[75] V. A. Q. Freitas , A. S. Santos , A. L. S. A. Zara , et al., “ *Lodderomyces elongisporus* as Causative Organism of Oropharyngeal Infections ‐ Two Case Reports,” Journal de Mycologie Médicale 34, no. 1 (2024): 101459. 38181627 10.1016/j.mycmed.2023.101459

[76] K. L. Daveson and M. L. Woods , “ *Lodderomyces elongisporus* Endocarditis in an Intravenous Drug User: A New Entity in Fungal Endocarditis,” Journal of Medical Microbiology 61, no. Pt 9 (2012): 1338–1340. 22683656 10.1099/jmm.0.047548-0

[77] S. R. Lockhart , S. A. Messer , M. A. Pfaller , and D. J. Diekema , “ *Lodderomyces elongisporus* Masquerading as *Candida parapsilosis* as a Cause of Bloodstream Infections,” Journal of Clinical Microbiology 46, no. 1 (2008): 374–376. 17959765 10.1128/JCM.01790-07 PMC2224306

[78] S. Ahmad , Z. U. Khan , M. Johny , et al., “Isolation of *Lodderomyces elongisporus* From the Catheter Tip of a Fungemia Patient in the Middle East,” Case Reports in Medicine 2013 (2013): 560406. 23653654 10.1155/2013/560406 PMC3638566

[79] M. C. Gutiérrez Galvis , E. M. Gil Güiza , M. A. Avellaneda Arciniegas , et al., “First Report of *Lodderomyces elongisporus* Identification in Intensive Care Unit Patients in Colombia,” American Journal of Infection Control 53, no. 9 (2025): 1011–1016. 40571060 10.1016/j.ajic.2025.06.018

[80] G. Butler , M. D. Rasmussen , M. F. Lin , et al., “Evolution of Pathogenicity and Sexual Reproduction in Eight *Candida* Genomes,” Nature 459, no. 7247 (2009): 657–662. 19465905 10.1038/nature08064 PMC2834264

[81] S. J. Priest and M. C. Lorenz , “Characterization of Virulence‐Related Phenotypes in *Candida* Species of the CUG Clade,” Eukaryotic Cell 14, no. 9 (2015): 931–940. 26150417 10.1128/EC.00062-15 PMC4551586

[82] N. Asai , Y. Shibata , A. Nakamura , et al., “Three Successfully Treated Cases of *Lodderomyces elongisporus* Fungemia: Case Reports and a Review of the Literature,” Microorganisms 11, no. 4 (2023): 1076. 37110499 10.3390/microorganisms11041076 PMC10142367

[83] O. Molnár , G. Schatzmayr , E. Fuchs , and H. Prillinger , “ *Trichosporon mycotoxinivorans* sp. nov., a New Yeast Species Useful in Biological Detoxification of Various Mycotoxins,” Systematic and Applied Microbiology 27, no. 6 (2004): 661–671. 15612623 10.1078/0723202042369947

[84] X. Z. Liu , Q. M. Wang , M. Göker , et al., “Towards an Integrated Phylogenetic Classification of the Tremellomycetes,” Studies in Mycology 81 (2015): 85–147. 26955199 10.1016/j.simyco.2015.12.001 PMC4777781

[85] D. Goldenberger , V. Hinić , S. S. Prince , et al., “A Case Report of a Cystic Fibrosis Patient With Repeated Isolation of *Trichosporon mycotoxinivorans* Identified by a Novel Short‐Extraction Method,” BMC Infectious Diseases 16, no. 1 (2016): 601. 27782810 10.1186/s12879-016-1910-7 PMC5078883

[86] P. W. Hickey , D. A. Sutton , A. W. Fothergill , et al., “ *Trichosporon mycotoxinivorans*, a Novel Respiratory Pathogen in Patients With Cystic Fibrosis,” Journal of Clinical Microbiology 47, no. 10 (2009): 3091–3097. 19656976 10.1128/JCM.00460-09 PMC2756937

[87] M. Chayakulkeeree and P. Chongtrakool , “Emergence of *Apiotrichum mycotoxinivorans* Invasive Infection in Thailand: Report of the First Four Cases in Southeast Asia,” International Journal of Infectious Diseases 152 (2025): 107568.

[88] X. Li , D. Wang , M. Hao , et al., “The First Report of *Apiotrichum mycotoxinivorans* Isolation From Human Cerebrospinal Fluid,” European Journal of Clinical Microbiology & Infectious Diseases 43, no. 3 (2024): 597–604. 38103075 10.1007/s10096-023-04736-0

[89] A. Kumar , P. Kumari , and M. Gaba , “Meningitis due to *Apiotrichum mycotoxinivorans*: A Rare Case Report,” Cureus 16, no. 9 (2024): e70573. 39483950 10.7759/cureus.70573 PMC11525039

[90] J. N. Almeida, Jr. , E. C. Francisco , M. G. M. A. Barberino , et al., “Emergence of *Trichosporon mycotoxinivorans* (*Apiotrichum mycotoxinivorans*) Invasive Infections in Latin America,” Memórias do Instituto Oswaldo Cruz 112, no. 10 (2017): 719–722. 28954000 10.1590/0074-02760170011 PMC5607521

[91] L. Peng , Y. Q. Jiang , G. M. Jiang , et al., “Molecular Identification and Biological Characteristic Analysis of an *Apiotrichum mycotoxinivorans* (Formerly *Trichosporon mycotoxinivorans*) Strain Isolated From Sputum Specimens of a Pediatric Patient With Pneumonia,” Journal de Mycologie Médicale 29, no. 2 (2019): 120–126. 30898449 10.1016/j.mycmed.2019.01.010

[92] C. Hsu and M. Yassin , “Diagnostic Approaches for *Candida auris*: A Comprehensive Review of Screening, Identification, and Susceptibility Testing,” Microorganisms 13, no. 7 (2025): 1461. 40731971 10.3390/microorganisms13071461 PMC12298640

[93] J. Zurita , A. Paz Y Miño , M. B. Solís , and G. Sevillano , “Failed Identification of *Candida vulturna* Using the Updated Vitek 2 Yeast Identification System, Version 9.02 and CHROMagar Candida Plus,” New Microbes New Infections 48 (2022): 101012. 36016725 10.1016/j.nmni.2022.101012 PMC9396391

[94] D. S. Hibbett , M. Binder , J. F. Bischoff , et al., “A Higher‐Level Phylogenetic Classification of the Fungi,” Mycological Research 111, no. Pt 5 (2007): 509–547. 17572334 10.1016/j.mycres.2007.03.004

[95] U. Binder , E. Maurer , and C. Lass‐Flörl , “Mucormycosis—From the Pathogens to the Disease,” Clinical Microbiology and Infection 20, no. Suppl 6 (2014): 60–66. 24476149 10.1111/1469-0691.12566

[96] A. Chakrabarti , R. S. Marak , M. R. Shivaprakash , et al., “Cavitary Pulmonary Zygomycosis Caused by *Rhizopus homothallicus* ,” Journal of Clinical Microbiology 48, no. 5 (2010): 1965–1969. 20200286 10.1128/JCM.01272-09 PMC2863885

[97] J. Taneja , V. K. Agrawal , D. Jamal , and Z. Abbas , “A Rare Case of Pulmonary Mucormycosis Caused by *Rhizopus homothallicus* in a Post Heart Transplant Patient,” Journal of Intensive and Critical Care 6 (2020): 23.

[98] S. M. Rudramurthy , S. Singh , R. Kanaujia , et al., “Clinical and Mycologic Characteristics of Emerging Mucormycosis Agent *Rhizopus homothallicus* ,” Emerging Infectious Diseases 29, no. 7 (2023): 1313–1322. 37347535 10.3201/eid2907.221491 PMC10310386

[99] H. Prakash and A. Chakrabarti , “Epidemiology of Mucormycosis in India,” Microorganisms 9, no. 3 (2021): 523. 33806386 10.3390/microorganisms9030523 PMC8000977

[100] J. Chander , N. Singla , M. Kaur , et al., “ *Apophysomyces variabilis*, an Emerging and Worrisome Cause of Primary Cutaneous Necrotizing Infections in India,” Journal de Mycologie Médicale 31, no. 4 (2021): 101197. 34454304 10.1016/j.mycmed.2021.101197

[101] K. Samaras , A. M. Markantonatou , D. Karapiperis , et al., “ *Saksenaea vasiformis* Infections: A Case of an Immunocompetent Adult After Mild Injury and a Literature Review,” Journal de Mycologie Médicale 29, no. 3 (2019): 260–264. 31445820 10.1016/j.mycmed.2019.06.005

[102] Z. Li , Q. Jiang , X. Tao , et al., “Primary Cutaneous Mucormycosis Caused by *Mucor irregularis* in a Chinese Man,” Mycopathologia 189, no. 3 (2024): 39. 38704804 10.1007/s11046-024-00844-3

[103] I. Xess , S. Mohapatra , M. R. Shivaprakash , et al., “Evidence Implicating *Thamnostylum lucknowense* as an Etiological Agent of Rhino‐Orbital Mucormycosis,” Journal of Clinical Microbiology 50, no. 4 (2012): 1491–1494. 22301030 10.1128/JCM.06611-11 PMC3318503

[104] F. Z. Li , M. Jia , A. J. Chen , and S. Fang , “Increase in the Incidence of Invasive Fungal Infections due to *Volvariella volvacea* ,” European Journal of Clinical Microbiology & Infectious Diseases 43, no. 5 (2024): 1031–1036. 38472521 10.1007/s10096-024-04800-3

[105] L. He , X. Mei , S. Lu , et al., “ *Talaromyces marneffei* Infection in Non‐HIV‐Infected Patients in Mainland China,” Mycoses 64, no. 10 (2021): 1170–1176. 34110649 10.1111/myc.13295

[106] J. F. W. Chan , S. K. P. Lau , K. Y. Yuen , and P. C. Y. Woo , “ *Talaromyces* (*Penicillium*) *marneffei* Infection in Non‐HIV‐Infected Patients,” Emerging Microbes & Infections 5, no. 3 (2016): e19. 26956447 10.1038/emi.2016.18 PMC4820671

[107] NIH , “Panel on Guidelines for the Prevention and Treatment of Opportunistic Infections in Adults and Adolescents With HIV,” in Guidelines for the Prevention and Treatment of Opportunistic Infections in Adults and Adolescents With HIV (National Institutes of Health, HIV Medicine Association, and Infectious Diseases Society of America, 2025), accessed August 2, 2026, https://clinicalinfo.hiv.gov/en/guidelines/adult‐and‐adolescent‐opportunistic‐infection.

[108] A. Atalay , A. N. Koc , G. Akyol , N. Cakir , L. Kaynar , and A. Ulu‐Kilic , “Pulmonary Infection Caused by *Talaromyces purpurogenus* in a Patient With Multiple Myeloma,” Le Infezioni in Medicina 24, no. 2 (2016): 153–157. 27367328

[109] H. Villanueva‐Lozano , R. J. Treviño‐Rangel , E. W. Renpenning‐Carrasco , and G. M. González , “Successful Treatment of *Talaromyces amestolkiae* Pulmonary Infection With Voriconazole in an Acute Lymphoblastic Leukemia Patient,” Journal of Infection and Chemotherapy 23, no. 6 (2017): 400–402. 28161296 10.1016/j.jiac.2016.12.017

[110] P. E. Santos , E. Piontelli , Y. R. Shea , et al., “ *Penicillium piceum* Infection: Diagnosis and Successful Treatment in Chronic Granulomatous Disease,” Medical Mycology 44, no. 8 (2006): 749–753. 17127632 10.1080/13693780600967089

[111] M. Guevara‐Suarez , D. A. Sutton , J. Gené , et al., “Four New Species of *Talaromyces* From Clinical Sources,” Mycoses 60, no. 10 (2017): 651–662. 28660627 10.1111/myc.12640

[112] S. S. Surja , R. Adawiyah , J. Houbraken , et al., “ *Talaromyces atroroseus* in HIV and Non‐HIV Patient: A First Report From Indonesia,” Medical Mycology 58, no. 4 (2020): 560–563. 31504774 10.1093/mmy/myz090

[113] A. Spallone and I. S. Schwartz , “Emerging Fungal Infections,” Infectious Disease Clinics of North America 35, no. 2 (2021): 261–277. 34016278 10.1016/j.idc.2021.03.014

[114] K. Vinayagamoorthy , D. R. Gangavaram , A. Skiada , and H. Prakash , “Emergomycosis, an Emerging Thermally Dimorphic Fungal Infection: A Systematic Review,” Journal of Fungi 9, no. 10 (2023): 1039. 37888295 10.3390/jof9101039 PMC10607913

[115] R. E. Mapengo , T. G. Maphanga , W. Grayson , and N. P. Govender , “Endemic Mycoses in South Africa, 2010‐2020: A Decade‐Long Description of Laboratory‐Diagnosed Cases and Prospects for the Future,” PLoS Neglected Tropical Diseases 16, no. 9 (2022): e0010737. 36170322 10.1371/journal.pntd.0010737 PMC9518919

[116] C. Ibe , P. N. Mnyambwa , and G. S. Mfinanga , “Emergomycosis in Africa: Time to Pay Attention to This Emerging Deadly Fungal Infection,” International Journal of General Medicine 16 (2023): 2313–2322. 37309324 10.2147/IJGM.S403797 PMC10257923

[117] A. Samaddar and A. Sharma , “Emergomycosis, an Emerging Systemic Mycosis in Immunocompromised Patients: Current Trends and Future Prospects,” Frontiers in Medicine (Lausanne) 8 (2021): 670731. 10.3389/fmed.2021.670731 PMC8104006 33968970

[118] I. S. Schwartz , N. P. Govender , L. Sigler , et al., “ *Emergomyces*: The Global Rise of New Dimorphic Fungal Pathogens,” PLoS Pathogens 15, no. 9 (2019): e1007977. 31536607 10.1371/journal.ppat.1007977 PMC6752945

[119] Y. Jiang , K. Dukik , J. F. Muñoz , et al., “Phylogeny, Ecology and Taxonomy of Systemic Pathogens and Their Relatives in Ajellomycetaceae (Onygenales): *Blastomyces*, *Emergomyces*, *Emmonsia*, *Emmonsiellopsis* ,” Fungal Diversity 90, no. 1 (2018): 245–291.

[120] R. Kano , U. Kimura , M. Kakurai , et al., “ *Trichophyton indotineae* sp. nov.: A New Highly Terbinafine‐Resistant Anthropophilic Dermatophyte Species,” Mycopathologia 185, no. 6 (2020): 947–958. 32449054 10.1007/s11046-020-00455-8

[121] B. Sonego , A. Corio , V. Mazzoletti , et al., “ *Trichophyton indotineae*, an Emerging Drug‐Resistant Dermatophyte: A Review of the Treatment Options,” Journal of Clinical Medicine 13, no. 12 (2024): 3558. 38930086 10.3390/jcm13123558 PMC11204959

[122] S. Prunglumpoo , K. Krongboon , K. Intarachaieua , et al., “Emergence of Resistant Dermatophytosis Caused by *Trichophyton indotineae*: First Case Series in Thailand,” Medical Mycology Case Reports 48 (2025): 100713. 40547227 10.1016/j.mmcr.2025.100713 PMC12179712

[123] S. Uhrlaß , S. B. Verma , Y. Gräser , et al., “ *Trichophyton indotineae*: An Emerging Pathogen Causing Recalcitrant Dermatophytoses in India and Worldwide—A Multidimensional Perspective,” Journal of Fungi (Basel) 8, no. 7 (2022): 757. 10.3390/jof8070757 PMC9323571 35887512

[124] L. Brescini , S. Fioriti , G. Morroni , and F. Barchiesi , “Antifungal Combinations in Dermatophytes,” Journal of Fungi 7, no. 9 (2021): 727. 34575765 10.3390/jof7090727 PMC8469868

[125] A. Chowdhary , H. S. Randhawa , S. N. Gaur , et al., “ *Schizophyllum commune* as an Emerging Fungal Pathogen: A Review and Report of Two Cases,” Mycoses 56, no. 1 (2013): 1–10. 10.1111/j.1439-0507.2012.02190.x 22524529

[126] C. Xue , J. Shen , J. Song , J. Yang , S. Zhang , and J. Wang , “Pulmonary Infection Caused by *Schizophyllum commune*: A Case Study,” Frontiers in Medicine (Lausanne) 12 (2025): 1475896. 10.3389/fmed.2025.1475896 PMC12206706 40589965

[127] J. Tschopp , J. Y. Perentes , C. Beigelman‐Aubry , et al., “Invasive *Hormographiella aspergillata* Infection in Patients With Acute Myeloid Leukemia: Report of Two Cases Successfully Treated and Review of the Literature,” Medical Mycology Case Reports 32 (2021): 68–72. 33996425 10.1016/j.mmcr.2021.03.008 PMC8095099

[128] R. B. Salit , Y. R. Shea , J. Gea‐Banacloche , et al., “Death by Edible Mushroom: First Report of *Volvariella volvacea* as an Etiologic Agent of Invasive Disease in a Patient Following Double Umbilical Cord Blood Transplantation,” Journal of Clinical Microbiology 48, no. 11 (2010): 4329–4332. 20826647 10.1128/JCM.01222-10 PMC3020845

[129] L. K. Chew , D. H. L. Ng , J. W. P. Teo , et al., “Disseminated *Volvariella volvacea* Infections in Patients With Haematological Malignancies: A Case Series,” Clinical Microbiology and Infection 25, no. 1 (2019): 117–119. 30099134 10.1016/j.cmi.2018.07.032

[130] K. Dukik , A. M. S. Al‐Hatmi , I. Curfs‐Breuker , D. Faro , S. de Hoog , and J. F. Meis , “Antifungal Susceptibility of Emerging Dimorphic Pathogens in the Family Ajellomycetaceae,” Antimicrobial Agents and Chemotherapy 62, no. 1 (2018): e01886‐17. 29084748 10.1128/AAC.01886-17 PMC5740316

[131] W. Xie , X. Kong , H. Zheng , et al., “ *In Vitro* Susceptibility Profiles of 16 Antifungal Drugs Against *Trichophyton indotineae* ,” Microbiology Spectrum 13, no. 8 (2025): e00618‐25. 40548712 10.1128/spectrum.00618-25 PMC12323362

[132] L. Mendoza and R. Vilela , “The Mammalian Pathogenic Oomycetes,” Current Fungal Infection Reports 7, no. 3 (2013): 198–208.

[133] M. B. Miraglia , L. Mendoza , R. Rammohan , et al., “ *Pythium insidiosum* Complex Hides a Cryptic Novel Species: *Pythium periculosum* ,” Fungal Biology 126, no. 5 (2022): 366–374. 35501032 10.1016/j.funbio.2022.03.002

[134] J. E. Sykes , ed., Greene's Infectious Diseases of the Dog and Cat, 5th ed. (Elsevier, 2023).

[135] H. Yolanda and T. Krajaejun , “Global Distribution and Clinical Features of Pythiosis in Humans and Animals,” Journal of Fungi 8, no. 2 (2022): 182. 35205934 10.3390/jof8020182 PMC8879638

[136] T. Krajaejun , B. Sathapatayavongs , R. Pracharktam , et al., “Clinical and Epidemiological Analyses of Human Pythiosis in Thailand,” Clinical Infectious Diseases 43, no. 5 (2006): 569–576. 16886148 10.1086/506353

[137] M. N. Chitasombat , P. Jongkhajornpong , K. Lekhanont , and T. Krajaejun , “Recent Update in Diagnosis and Treatment of Human Pythiosis,” PeerJ 8 (2020): e8555. 32117626 10.7717/peerj.8555 PMC7036273

[138] F. Ros Castellar , C. Sobrino Jiménez , A. del Hierro Zarzuelo , A. Herrero Ambrosio , and A. Boto de Los Bueis , “Intraocular Minocycline for the Treatment of Ocular Pythiosis,” American Journal of Health‐System Pharmacy 74, no. 11 (2017): 821–825. 28546303 10.2146/ajhp160248

[139] V. Puangsricharern , P. Chotikkakamthorn , W. Tulvatana , et al., “Clinical Characteristics, Histopathology, and Treatment Outcomes of *Pythium* Keratitis: A Retrospective Cohort Study,” Clinical Ophthalmology 15 (2021): 1691–1701. 33935486 10.2147/OPTH.S303721 PMC8080432

[140] N. Nutthapan , W. Sutichaiworapong , and Y. H. Wu , “Human Cutaneous Pythiosis: A Case Report,” Journal of Cutaneous Pathology 51, no. 12 (2024): 964–968. 39266281 10.1111/cup.14719

[141] N. Permpalung , N. Worasilchai , and A. Chindamporn , “Human Pythiosis: Emergence of Fungal‐Like Organism,” Mycopathologia 185, no. 5 (2020): 801–812. 31845178 10.1007/s11046-019-00412-0

[142] L. Mendoza and J. Prendas , “A Method to Obtain Rapid Zoosporogenesis of *Pythium insidiosum* ,” Mycopathologia 104, no. 1 (1988): 59–62. 3216883 10.1007/BF00437925

[143] P. Torvorapanit , N. Worasilchai , K. Manothummetha , et al., “Improved Survival in Vascular Pythiosis With Surgery and Azithromycin, Doxycycline, and Itraconazole Therapy: A Phase II Multicenter, Open‐Label, Single‐Arm Trial,” Clinical Infectious Diseases 80, no. 6 (2025): 1281–1289. 39932261 10.1093/cid/ciaf062

[144] A. M. Grooters , “Pythiosis, Lagenidiosis, and Zygomycosis in Small Animals,” Veterinary Clinics of North America. Small Animal Practice 33, no. 4 (2003): 695–720. 12910739 10.1016/s0195-5616(03)00034-2

[145] J. Theophilopoulos , R. King , A. Citta , et al., “Cutaneous *Lagenidium deciduum* Infection in a Patient With Relapsed Acute Myeloid Leukemia,” BMC Infectious Diseases 24, no. 1 (2024): 515. 38778275 10.1186/s12879-024-09281-5 PMC11112786

[146] U. Reinprayoon , N. Permpalung , N. Kasetsuwan , R. Plongla , L. Mendoza , and A. Chindamporn , “ *Lagenidium* sp. Ocular Infection Mimicking Ocular Pythiosis,” Journal of Clinical Microbiology 51, no. 8 (2013): 2778–2780. 23740721 10.1128/JCM.00783-13 PMC3719631

[147] N. P. Wiederhold , “Antifungal Susceptibility Testing: A Primer for Clinicians,” Open Forum Infectious Diseases 8, no. 11 (2021): ofab444. 34778489 10.1093/ofid/ofab444 PMC8579947

[148] C. Lass‐Flörl , M. Lass , J. M. Kern , and S. Huber , “Antifungal Susceptibility Testing Across Fungi: Why MICs Vary and Methods Diverge and What MIC Can Miss,” Clinical Microbiology and Infection 32, no. 8 (2026): 1260–1264. 41825586 10.1016/j.cmi.2026.03.004

